# Sex Does Matter! The Influence of Sex on Outcomes of Transcranial Direct Current Stimulation (tDCS)

**DOI:** 10.3390/cells15161474

**Published:** 2026-08-17

**Authors:** James Chmiel, Marta Stępień-Słodkowska, Aleksandra Kładna, Mirela Niedzielska

**Affiliations:** 1 Faculty of Physical Culture and Health, Institute of Physical Culture Sciences, University of Szczecin, Al. Piastów 40B Block 6, 71-065 Szczecin, Poland; marta.stepien-slodkowska@usz.edu.pl; 2 Department of the History of Medicine and Medical Ethics, Pomeranian Medical University, ul. Rybacka 1, 70-204 Szczecin, Poland

**Keywords:** tDCS, transcranial direct current stimulation, sex, gender, non-invasive brain stimulation, neurostimulation, neuromodulation

## Abstract

**Background:** Transcranial direct current stimulation (tDCS) produces heterogeneous outcomes across cognitive, behavioural, and clinical studies. Sex-related differences may contribute to this variability. **Objective:** This narrative review examined whether and how sex moderates tDCS outcomes across behavioural, physiological, clinical, and modelling studies and considered potential underlying mechanisms. **Materials and Methods:** A structured literature search identified studies examining sex, or variables reported by the original authors as gender but operationalised through female–male group comparisons, as moderators of tDCS outcomes. After screening, 41 studies met the inclusion criteria, and 6 additional studies were identified through citation searching, yielding 47 studies. Because of substantial heterogeneity in populations, protocols, and outcomes, findings were synthesised narratively. **Results:** Sex-related effects were common but highly context-dependent, varying by montage, cortical target, stimulation intensity, reference placement, and outcome domain. They often emerged as interaction effects rather than main effects and were most apparent during demanding tasks, later learning phases, or delayed after-effects. Computational models indicated that identical stimulation settings may produce different intracranial electric fields in women and men because of anatomical differences. Hormonal and endocrine states, particularly in females, may further modify or amplify these effects. Sensation, tolerability, and blinding may also contribute under higher-intensity protocols but do not fully explain sex-contingent outcomes. **Conclusions:** Sex influences tDCS outcomes through interacting anatomical, hormonal, and task-related mechanisms rather than in a uniform manner. Future studies should treat sex as a mechanistically relevant moderator, incorporate dose- and hormone-aware designs, and use adequately powered analyses to improve reproducibility and support individualised neuromodulation.

## 1. Introduction

Transcranial direct current stimulation (tDCS) is a non-invasive technique used to temporarily modulate activity in selected brain regions [1]. It is generally considered painless, safe, easy to tolerate, and associated with minimal or no side effects [2,3]. The method works by passing a weak, constant electrical current (1–3 mA) between two electrodes positioned on the scalp. This current changes the resting membrane potential, which can either enhance cortical excitability through anodal stimulation or reduce it through cathodal stimulation [4,5], with effects lasting up to 90 min after stimulation ends. In broad terms, cathodal stimulation tends to hyperpolarise neurons and make them less likely to fire, whereas anodal stimulation depolarises neurons and increases their excitability [6]. The exact outcome, however, also depends on how neuronal structures are oriented within the stimulated cortical area. As the electrical current flows between electrodes, tDCS is not highly focal by nature, though targeting can be improved by modifying electrode size [7]. Its neuroplastic effects can become stronger and longer-lasting when stimulation is applied repeatedly. At the mechanistic level, tDCS engages calcium-dependent synaptic plasticity in glutamatergic neurons through N-methyl-D-aspartate (NMDA) receptors. These processes produce either long-term depression (LTD) or long-term potentiation (LTP) depending on intracellular conditions [8,9]. Applying 1–2 mA for longer than 10 min can stabilise these synaptic effects for at least an hour, while even brief stimulation periods of around 3 min may produce effects that persist beyond the stimulation itself [5]. In addition, tDCS affects neurotransmitter systems by modulating glutamate, lowering gamma-aminobutyric acid (GABA), and influencing brain-derived neurotrophic factor (BDNF). Together, these effects help rebalance cortical excitation and inhibition [10,11,12,13]. Importantly, these effects may spread beyond the directly stimulated area to other functionally connected brain networks [14].

Across the broader tDCS literature, multiple reviews converge on the conclusion that robust effects are not uniformly observed and that null or inconsistent findings are common. This is especially evident when protocols are applied as “one-size-fits-all” interventions and evaluated with distal behavioural endpoints. In healthy cognition, a widely cited quantitative review of single-session studies concluded that reliable cognitive effects are not evident across domains, arguing that many purported enhancements do not replicate consistently when the literature is aggregated [15]. At the same time, other meta-analytic work illustrates that effects can be domain- and outcome-specific rather than global. For example, single-session stimulation shows more reliable effects in some language measures than in broad “cognition” composites, highlighting that what looks like “tDCS works/doesn’t work” often depends on which function and which metric is analysed [16]. This heterogeneity is not treated as a minor nuisance in modern reviews. Instead, it is increasingly framed as a core feature of transcranial electrical stimulation. It is driven by parameter sensitivity (montage, intensity, duration, reference placement, timing relative to task engagement) and by strong state dependence. As a result, identical stimulation can yield different outcomes depending on ongoing neural activity and baseline performance [13,17,18]. Reviews of individual differences also demonstrate substantial variability in tDCS responsiveness. Anatomy, baseline excitability, age, and other biological factors may determine whether a nominal dose adequately engages the intended target. Consequently, null group-level effects may coexist with meaningful effects in specific subgroups or task states [19,20]. Clinical syntheses reinforce the same theme: in depression, systematic reviews and meta-analyses commonly describe mixed results and modest average benefits, with outcomes varying across trials as a function of protocol choices, trial design, and patient characteristics—again consistent with a treatment whose efficacy is conditional rather than universal [21,22,23].

The aim of this review is to examine whether and how sex moderates tDCS outcomes. Specifically, it synthesises studies that compared female and male participants, tested sex-by-stimulation interactions, examined sex-related anatomical differences in delivered electric fields, or investigated hormonal and endocrine factors relevant to sex-linked variability. Gender identity was not directly assessed in the available literature and is therefore not evaluated as an independent moderator in this review. Sex-related variability in tDCS should not be treated merely as demographic noise. Instead, it may reflect biologically meaningful differences in skull and cerebrospinal fluid geometry, cortical folding, endocrine state, and neurochemical regulation of excitability and plasticity. These factors are directly relevant to how electrical current reaches cortical tissue and how neuronal populations respond once stimulated. Examining sex-related effects therefore provides a route to understanding mechanistic determinants of dose delivery and variability in neuromodulation outcomes. This review summarises the characteristics of the included studies, synthesises findings across outcome domains, discusses potential mechanisms, and outlines limitations and directions for future research.

**Figure 1:**
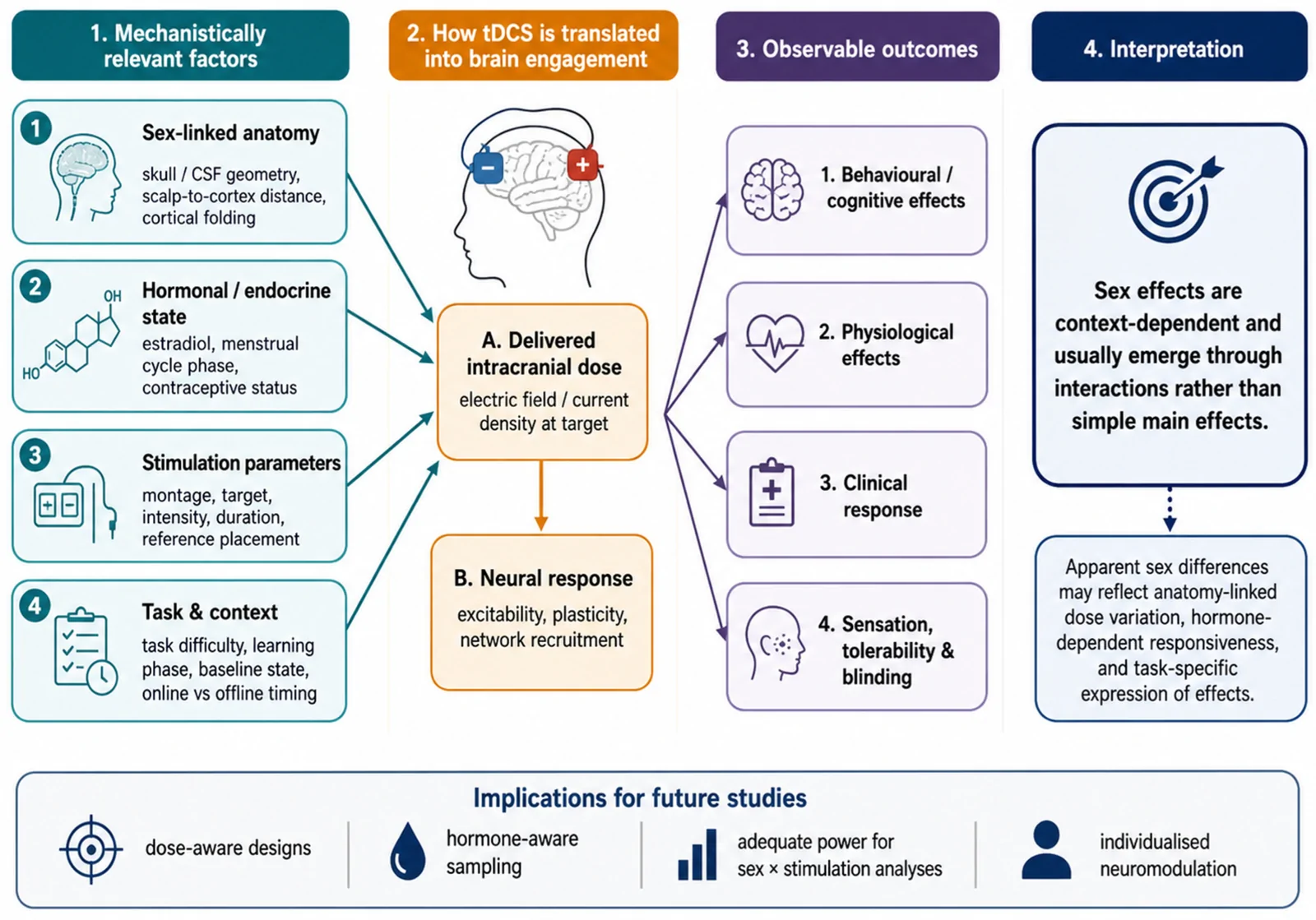
Conceptual model of sex-related variability in tDCS outcomes. Sex-related differences in tDCS response are proposed to arise from the interaction between sex-linked anatomy, hormonal/endocrine state, stimulation parameters, and task context. These factors can influence the delivered intracranial dose, including electric field strength and current density at the target, which then shapes neural responses such as cortical excitability, plasticity, and network recruitment. Observable outcomes may therefore differ across behavioural/cognitive, physiological, clinical, and sensation/tolerability domains. The figure emphasises that sex effects in tDCS are usually context-dependent and often emerge through interactions rather than simple male–female main effects.

To provide a conceptual framework for the review, Figure 1 summarises the proposed model of sex-related variability in tDCS outcomes, showing how sex-linked anatomy, hormonal/endocrine state, stimulation parameters, and task context may influence delivered intracranial dose, neural response, and observable behavioural, physiological, clinical, and tolerability outcomes.

## 2. Materials and Methods

This review was conducted as a narrative review with a structured literature search and study-selection process. The aim was to identify and synthesise studies examining whether sex moderated tDCS outcomes, including behavioural, physiological, clinical, and computational-modelling outcomes. Studies using the term “gender” were eligible when the variable was operationalised through comparisons between female/women and male/men participants; however, because gender identity was not directly assessed, these studies were interpreted as examining sex-related rather than gender-related differences.

### 2.1. Search Strategy

A literature search was performed to identify original studies on tDCS that evaluated sex-related differences in stimulation effects. The term “gender” was retained in the search strategy because it is frequently used in the older and current tDCS literature as a synonym for sex; inclusion of this term was intended to maximise retrieval and does not indicate that the review treated sex and gender as conceptually equivalent. The search was designed to capture studies across both healthy and clinical populations and across a broad range of outcomes (e.g., cognition, motor performance, symptoms, neurophysiology, and computational modelling of current flow).

After the initial search and screening, the reference lists of eligible articles were examined. Similar, cited, and citing articles were also checked to identify additional relevant studies.

The literature search was conducted in PubMed, Scopus, and Web of Science from database inception to 20 February 2026. Search terms combined transcranial direct current stimulation-related terms (“tDCS” OR “transcranial direct current stimulation”) with sex-related terms (“sex”, “gender”, “female”, “male”, “women”, “men”, “hormonal”, “menstrual cycle”, “estradiol”). Searches were limited to peer-reviewed human studies published in English. Titles and abstracts were screened first, followed by full-text review of potentially relevant records. Reference lists of eligible papers, as well as cited and citing articles, were also examined to identify additional studies. Because of the heterogeneity of populations, stimulation protocols, and outcome measures, the review was planned as a structured narrative synthesis rather than a formal systematic review or meta-analysis.

### 2.2. Eligibility Criteria

Studies were included if they:

- Used transcranial direct current stimulation (tDCS) (including conventional and HD-tDCS approaches);
- Reported outcomes in humans (behavioural, physiological, clinical, or modelling-based);
- Explicitly examined sex/gender as a moderator, grouping variable, interaction term, or biologically relevant factor influencing tDCS effects (including studies focused on hormonal state within women when relevant to sex-linked variability). Explicitly examined **sex** as a moderator, grouping variable, interaction term, or biologically relevant factor influencing tDCS effects. Studies describing the variable as “gender” were included only when it was operationalised through female–male or women–men comparisons. Studies examining hormonal or menstrual-cycle state within women were also included when relevant to sex-linked variability.

Studies were excluded if they:

- Used tDCS but did not analyse sex-related effects or female–male group differences; Used tDCS but did not analyse sex/gender effects;
- Did not report outcomes relevant to the review aim;
- Fell outside the scope of human tDCS research.

### 2.3. Data Extraction and Synthesis

For each included study, the following information was extracted and summarised:

Study design and population (healthy/clinical; sample characteristics);

- tDCS protocol (target, montage, intensity, duration, reference placement);
- Outcome domain (behavioural, physiological, clinical, modelling);
- How sex was operationalised, including whether the original study labelled the variable as “sex” or “gender,” how female and male groups were classified, and whether hormonal status, menstrual-cycle phase, contraceptive use, or gender identity was assessed; how sex/gender was operationalised (e.g., male/female group comparisons, sex × stimulation interactions, hormonal/cycle-related analyses);
- And the main findings regarding sex/gender moderation.

For studies involving menstrual-cycle phase or hormonal contraception, we additionally extracted whether endocrine status was established through direct hormone assays, cycle-day estimation, menstrual-history reporting, ovulation testing, ultrasonographic verification, or contraceptive-use status. We distinguished studies that directly measured circulating estradiol or progesterone from those that used menstrual-cycle phase as an indirect proxy for the broader endocrine milieu. Hormonal-contraceptive exposure was also treated cautiously because contraceptive preparations differ in oestrogen dose, progestin type, androgenic or anti-androgenic activity, administration schedule, and resulting suppression of endogenous ovarian hormones. Where these details were not reported, hormonal contraception was considered an incompletely characterised exposure rather than a unitary biological condition.

Substantial heterogeneity in study designs, stimulation parameters, populations, and outcome measures meant that a quantitative meta-analysis was inappropriate. Findings were therefore synthesised narratively, with emphasis on recurring patterns and sources of heterogeneity (e.g., montage, intensity, task demands, timing, and biological factors).

### 2.4. Terminology

In this review, sex refers to biological attributes and sex-related physiological characteristics, including anatomy, endocrine function, hormonal status, and neurophysiological processes. Gender refers to socially and culturally shaped identities, roles, behaviours, and experiences. Although several included publications used the term “gender,” the reviewed studies generally classified participants as female/women or male/men without directly assessing gender identity, gender roles, or other psychosocial dimensions of gender. Consequently, the available evidence does not permit conclusions about gender identity as a moderator of tDCS outcomes.

Throughout this review, we therefore use sex and sex-related differences when describing the variables analysed in the included studies and the anatomical, hormonal, physiological, or neurobiological mechanisms proposed to underlie them. The term gender is retained only when reporting the terminology used in the title or text of an original publication, or when explicitly discussing gender as a construct that was not measured in the available literature. Terms such as “women” and “men” are used when describing the participant groups reported by the original studies, but these classifications should not be interpreted as evidence that gender identity was assessed. Accordingly, this review examines sex as an operationalised study variable and does not evaluate the independent influence of gender identity on tDCS responsiveness.

### 2.5. Interpretation of Menstrual-Cycle and Hormonal Variables

Estradiol and progesterone fluctuate jointly across the menstrual cycle and should not be interpreted as independent phase markers. The late follicular and periovulatory phases are generally characterised by rising or high estradiol with comparatively low progesterone, whereas the luteal phase is characterised by elevated progesterone together with moderate or secondary elevations in estradiol. Consequently, a comparison between follicular and luteal phases cannot by itself establish that an observed difference was caused specifically by estradiol or progesterone. Unless circulating hormone concentrations were directly assayed, we therefore refer to menstrual-cycle phase, cycle-related endocrine state, or the estradiol–progesterone milieu, rather than attributing findings to a single hormone. Hormone-specific interpretations are presented as mechanistic hypotheses and not as demonstrated causal explanations.

## 3. Results

**Figure 2:**
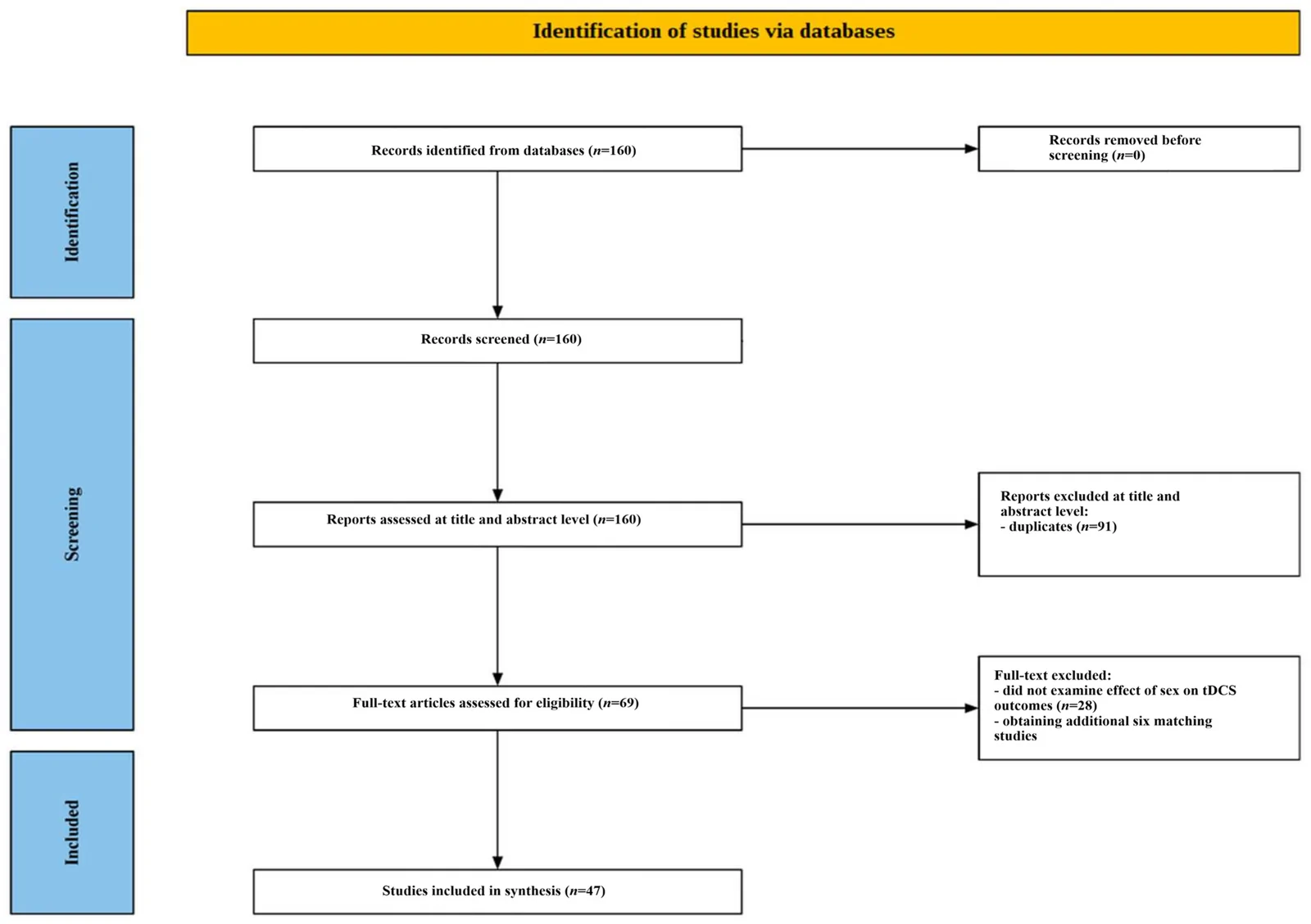
Flow chart depicting the different phases of the narrative review.

The search and selection process for studies is presented in Figure 2. The literature search identified 160 studies. After removing duplicates (*n* = 91), 69 studies were included in the full-text review. At this stage, 28 studies were excluded because they did not examine the effect of gender on tDCS outcomes. Studies that fit the scope of the review (*n* = 41) were searched for similar, cited, and citing articles. This resulted in six additional relevant studies being identified. Ultimately, 47 studies were included in the review [24,25,26,27,28,29,30,31,32,33,34,35,36,37,38,39,40,41,42,43,44,45,46,47,48,49,50,51,52,53,54,55,56,57,58,59,60,61,62,63,64,65,66,67,68,69,70].

**Figure 3:**
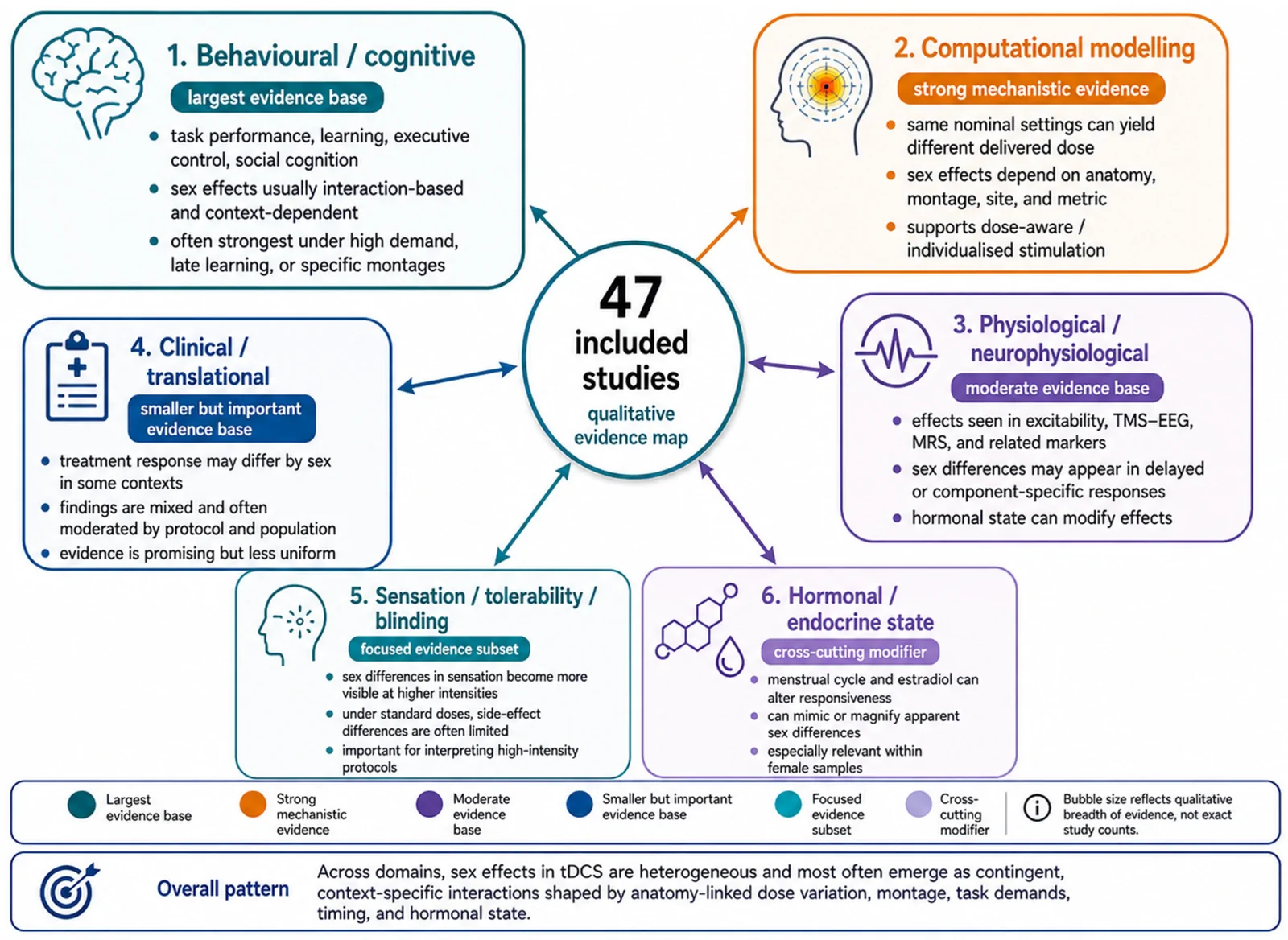
Qualitative outcome-domain map of the included evidence. The 47 included studies are organised according to the main domains in which sex-related tDCS effects were examined: behavioural/cognitive outcomes, computational modelling, physiological/neurophysiological outcomes, clinical/translational outcomes, sensation/tolerability/blinding, and hormonal/endocrine state. Bubble size reflects the qualitative breadth of evidence rather than exact study counts. The map shows that the largest evidence base concerns behavioural and cognitive outcomes, whereas modelling studies provide strong mechanistic support for dose-related interpretations and hormonal studies act as a cross-cutting modifier of apparent sex effects. This structure follows the review’s narrative synthesis of 47 included studies.

The distribution of the included evidence across outcome domains is summarised in Figure 3, which maps the 47 studies according to behavioural/cognitive, computational modelling, physiological/neurophysiological, clinical/translational, sensation/tolerability/blinding, and hormonal/endocrine dimensions.

### 3.1. Study Types and Operationalisation of Sex-Related Effects

Across the included literature, the terminology used by original authors was inconsistent. Some studies referred to “sex,” whereas others used “gender,” although nearly all operationalised the variable as a binary comparison between female/women and male/men participants. None of the included studies directly measured gender identity as a moderator of tDCS response. For consistency and conceptual accuracy, the findings are therefore described below as sex-related effects, regardless of the terminology used in the original publication.

Across the included studies, “sex effects” were operationalised in several distinct ways, and this distinction strongly shapes interpretation. One group of studies focused on whether the delivered biophysical dose differs by sex when the device settings are held constant, using individualised head models and finite-element simulations of current density or electric field (EF) (e.g., [47,48,51,56], and the modelling component embedded in [42]). A second cluster asked whether behavioural/physiological outcomes following tDCS differ by sex, typically via sex × stimulation interactions in sham-controlled designs (e.g., [26,30,31,35,36,37,38,39,40,41,42,43,44,45,46,47,48,49,50,51,52,53,54,55,56,57,58,59,60,61,62,63,64,65,66]) or via hormone state within women as a mechanistic modifier that can mimic or amplify “sex differences” (e.g., [27,32], and cycle controls in [38]). A third group, primarily clinical, investigated sex as a moderator of treatment response trajectories in applied or real-world contexts (e.g., tinnitus clinic outcomes including an HD-tDCS arm in [29], and network-connectivity changes in PPA therapy paired with tDCS in [41]). Importantly, Ref. [29] expands the ‘result types’ in this review beyond modelled dose and lab-task performance. It uses a clinic-facing symptom endpoint (TFI change and clinically meaningful response thresholds). It also embeds sex moderation in a multi-treatment, non-randomised care pathway rather than a tightly controlled single-task experiment. Several additional study types broadened the evidence base. One study examined menstrual-cycle phase as a modifier of sex-linked outcomes in a parietal attention task [64]. Another tested whether parietal tDCS could reduce a male–female performance gap in mental rotation [65]. Three clinical or translational studies evaluated sex alongside other potential moderators in post-stroke aphasia, schizophrenia, and depression [66,67,68]. Two additional social–economic studies targeted right TPJ to test whether “fairness-relevant” stimulation effects are sex contingent in inequity choices [69] and in sensitivity to CEO pay-ratio disclosures in investment judgments [70].

This diversity is critical for interpretation. A sex difference in modelled dose does not necessarily translate into a difference in behavioural or clinical outcomes. Several studies show that both the presence and direction of sex effects depend on montage, target, age, task domain, or hormone state [24,25,28,33,41,42].

### 3.2. Stimulation Intensity, Dose Scaling, and Individualised Dosing

Across the included modelling and behavioural studies, “dose” has two related meanings. The first refers to the nominal device settings, including current intensity, duration, electrode size, and montage. The second refers to the resulting intracranial field or current actually delivered to the target tissue. Where modelling studies varied intensity systematically, the most consistent—and reassuring—baseline result is near-linear scaling: doubling applied current roughly doubled modelled current density/EF under the target ROI within a given montage and pipeline [24,25]. This distinction is critical because many reported sex differences are not failures of scaling per se. Instead, they reflect differences in effective dosing at the same nominal intensity. In other words, they arise from different intercepts and interaction patterns that depend on montage, site, and anatomy [24,25].

Behavioural evidence suggests that sex moderation is often easiest to detect at higher intensities. At these levels, stimulation approaches thresholds where discomfort, peripheral co-stimulation, or nonlinear neurophysiological responses may become behaviourally visible. In leg fatiguability, the sex × stimulation interaction was driven specifically by the 4 mA condition: women showed greater extensor fatiguability than men at 4 mA, whereas 2 mA did not produce the same separation [30]. Complementary tolerability work similarly found that subjective sensation severity increased with intensity overall, and that the escalation with intensity was especially pronounced in women—again making 4 mA the most sex-differentiating condition [34]. Importantly, high-intensity effects can also reflect endocrine state rather than sex per se. In a 4 mA protocol restricted to women, active stimulation worsened extensor fatiguability during sessions classified by the investigators as having higher estradiol levels, whereas little or no active–sham separation was observed during lower-estradiol sessions [27]. It is because ovarian hormones covary across the menstrual cycle that this finding should not be interpreted as proving an isolated causal effect of estradiol unless progesterone and other relevant endocrine variables were simultaneously measured and modelled. It is more conservatively interpreted as evidence that the cycle-related hormonal milieu modified responsiveness to high-intensity stimulation. Together, these findings place intensity as a practical “amplifier”. Sex-linked differences may be subtle or absent at conventional doses. However, they can emerge at higher intensities or under specific physiological contexts that alter baseline excitability and peripheral sensations [27,30,34].

At the same time, sex moderation is not confined to an extreme dose window. Across the broader set, sex-dependent behavioural effects were reported under conventional 1–2 mA protocols delivered for roughly 6–25 min in both cognitive and social–economic paradigms, typically as interaction-structured effects rather than uniform sex main effects (e.g., context- or subgroup-gated stimulation × sex terms) [44,45,46,57,58,61,62,64,65,69,70]. Newer studies remain largely within standard parameters (e.g., ~1.5–2 mA for ~15–20 min with cheek/arm references in PPC/TPJ paradigms, and 2 mA multi-session bifrontal protocols in clinical samples), indicating that detectable sex moderation can arise even when dosing is well within common safety and comfort ranges [64,65,67,69,70]. In contrast, a large safety-focused trial varied intensity from 0 to 2 mA across diverse montages. It found moderate Bayesian evidence for no meaningful sex difference in tolerability attributable to stimulation. Any observed sex effects were localised to baseline symptom reporting rather than stimulation-induced side effects [60]. This supports a useful distinction for interpretation: sex moderation appears more robust and heterogeneous for efficacy/behavioural endpoints than for short-term tolerability under typical parameters [44,45,46,57,58,60].

A key mechanistic bridge between nominal dosing and apparent “sex effects” comes from individualised dosing. Under fixed-dose stimulation targeting left IPL, sex moderated behavioural change as a function of modelled current density at the target, with women and men showing divergent estimated effects at a given density. When current amplitude was individualised to equalise modelled target current density, sex was no longer associated with percentage reaction time change. Women and men showed similar estimated improvements. This implies that some sex-linked response differences under fixed dosing can be reduced by model-informed dose control [55]. This dovetails with modelling evidence that identical device settings can yield sex-linked differences in delivered cortical dose under specific montages and metrics [54], and reframes part of the literature as a dosing problem: sex-linked anatomy → different delivered dose at the target → apparent sex moderation in outcomes. Put simply, intensity and duration define the nominal intervention, but sex moderation is often best interpreted through the lens of delivered intracranial dose—especially when individualised modelling can directly test whether equalising target engagement attenuates sex associations [24,25,54,55].

**Figure 4:**
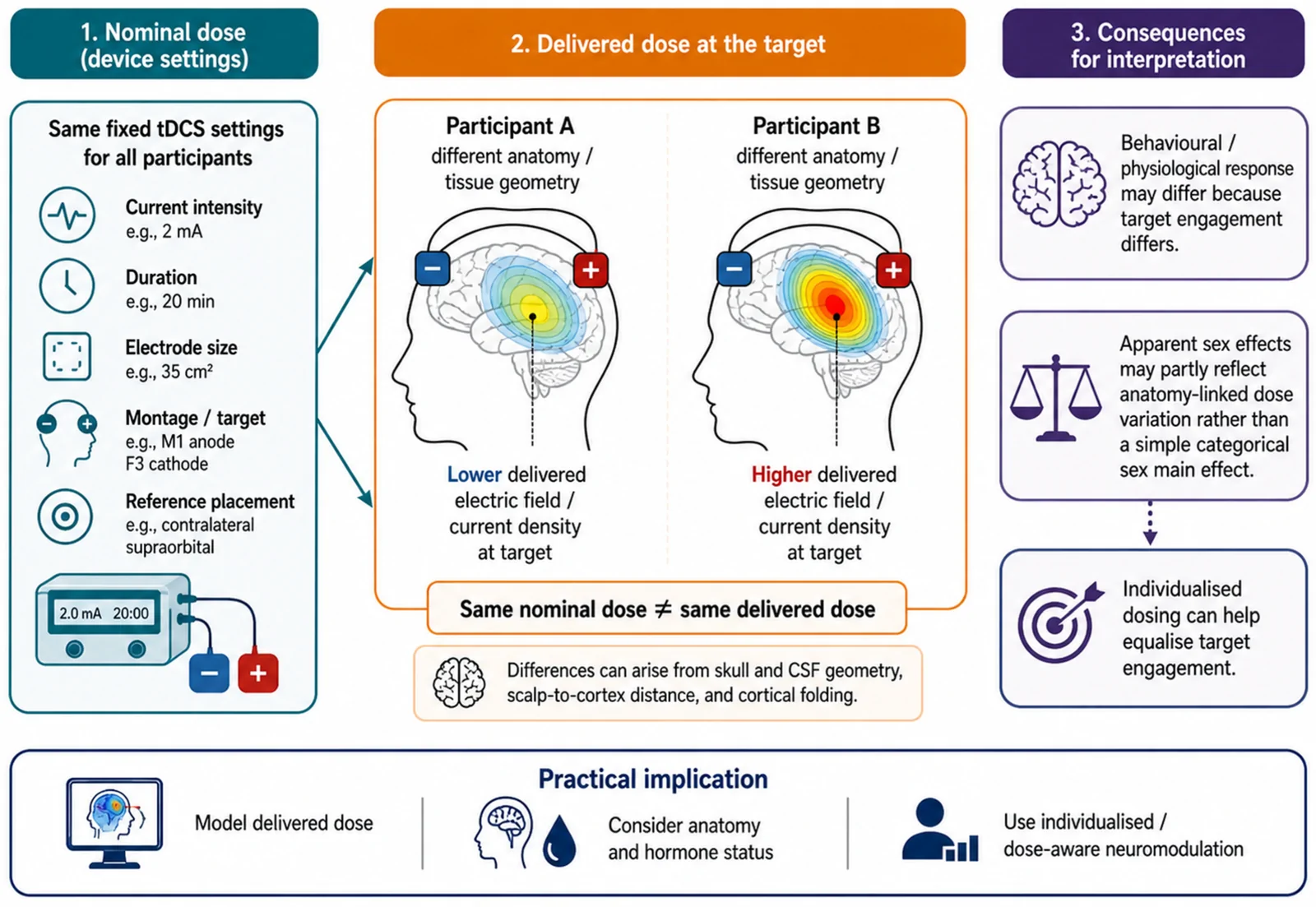
Nominal dose versus delivered intracranial dose in tDCS. The same nominal tDCS protocol, defined by current intensity, duration, electrode size, montage, target, and reference placement, may produce different electric fields or current densities at the cortical target across individuals. Anatomical differences in skull and cerebrospinal fluid geometry, scalp-to-cortex distance, tissue conductivity, and cortical folding can alter how much current reaches the intended brain region. As a result, identical device settings do not necessarily produce equivalent biological stimulation, and apparent sex-related effects may partly reflect differences in delivered dose rather than sex as a simple categorical variable.

This distinction between nominal stimulation parameters and biologically delivered dose is illustrated in Figure 4, which shows that identical device settings may produce different intracranial electric fields or current densities at the target because of individual anatomical and physiological variability.

#### Individual Variability and Weak Cross-Site Reliability in Delivered Dose

A second modelling result that cuts across studies is the sheer magnitude of individual variability under identical device settings. Both Ref. [24] and Ref. [25] show multi-fold spreads in modelled cortical current density/EF at the same nominal mA, implying that ‘dose’ varies strongly across people even when protocols are standardised. Importantly, Ref. [24] also reports weak within-person correspondence across scalp sites (C3 vs. F3 correlations only ~0.1–0.3), meaning that a person who receives relatively high modelled dose at one target may not receive relatively high dose at another. This weak cross-site reliability suggests that anatomical factors influencing current flow are not globally expressed, but can be location- and montage-specific—one reason why sex effects and behavioural outcomes may not generalise cleanly across targets [24,25].

### 3.3. Montage Geometry, Target Location, and Reference Placement as Primary Determinants of Sex Moderation

Across both modelling and behavioural studies, whether “sex effects” appear—and even their direction—depends strongly on how current is delivered: the target site, electrode geometry (size/focality), and where the return electrode is placed. In computational work, sex differences in modelled dose are not uniform. Instead, they are consistently configuration- and location-dependent. One study found males had higher modelled current density than females overall, but the difference was strongest in a ring-at-C3 reference condition and shifted across configurations and at F3, yielding explicit sex × configuration and sex × location interactions [24]. Another modelling study reported a clear parietal sex effect (men > women). However, it found no significant frontal sex effect under their averaging scheme [25]. Extending this across the lifespan, a large MRI-based simulation showed that sex effects on target current density depend on montage and age. Age × sex interactions produced male > female differences in young adults for a parietal montage. These patterns were weaker or reversed in older adults. Frontal montage effects were generally weaker and more age-driven [33]. These modelling results jointly argue that sex-linked anatomy does not impose a single global dosing offset; instead, it interacts with montage and site to shape which tissues and pathways dominate current flow [24,25,33].

Electrode geometry adds an additional—and sometimes dominant—layer. In one modelling framework, montage choice produced order-of-magnitude differences in cortical current density (e.g., non-cephalic > bi-cranial > ring/high-density as implemented there), with sex acting as an additional multiplicative factor layered onto configuration effects [24]. Electrode size can also behave counter to simple heuristics about “focality”: a study comparing a small round electrode to a larger rectangular electrode found the larger electrode produced higher modelled cortical current densities at ROIs, and sex differences (men > women) emerged for parietal but not frontal stimulation after averaging across hemispheres and sizes [25]. Not all pipelines converge on the same sex direction, however: an individualised modelling study using a standard C3–supraorbital montage reported higher mean/median whole-brain EF in females than males (~10–11% higher) with similar peak EF, implying a distributional shift rather than a peak-driven effect [28]. Together with age-dependent reversals [33], these findings support a conservative synthesis. Sex differences in delivered EF/current are montage-, metric-, and pipeline-sensitive. This includes differences between target-ROI current density and whole-brain EF summaries, as well as between current density and EF magnitude. These patterns reflect different combinations of skull composition, tissue proportions, and current-flow pathways emphasised by each setup [24,25,28,33]. Modelling embedded within a behavioural rOFC study similarly suggested deeper/higher EF in a representative female model for that montage, reinforcing that montage-specific anatomy can favour one sex in effective dosing under identical device settings [42].

Behavioural studies mirror this dependence, but in outcomes rather than fields: sex moderation frequently appears as sex × montage × target contingencies. In sensorimotor HD-tDCS, sex moderated a specific reaction-time effect under M1 stimulation, while other changes were sex-dependent but not stimulation-site specific, and some tactile findings were fragile once sex was modelled as a covariate [26]. In executive-control paradigms targeting DLPFC, sex did not moderate the primary stopping metric (SSRT) but did moderate how stimulation shaped practice-related Go accuracy changes [31]. A working-memory study further showed montage- and demand-dependent sex moderation: at the highest load in a high-interference verbal WM task, men benefited most from left DLPFC anodal stimulation relative to sham, whereas women showed the strongest benefit under right DLPFC stimulation, with a direct left-vs-right divergence within women [39]. Social-cognitive and affective domains show similarly site-specific patterns: anodal mPFC stimulation sped intention attribution in women but not men and was not reproduced by an anodal vertex control montage [36]; a bilateral temporal montage produced opposite error-direction effects for sad faces in women versus men [37]; and in a cross-modal sound–shape go/no-go task, sex moderation emerged only when polarity and congruency were considered together (a congruency × stimulation × gender interaction for NoGo accuracy), underscoring that montage can reveal sex effects as qualitatively different response directions rather than just different magnitudes [40]. Parietal paradigms likewise illustrate that even within the same broad region, hemisphere and endpoint can gate sex moderation: left-parietal anodal stimulation improved computerised midpoint-recognition accuracy primarily in men while leaving manual bisection unchanged across sexes and cycle phases [64]; in mental rotation, right-PPC stimulation most consistently improved accuracy/speed and eliminated a sham-condition gender gap, while left-PPC stimulation preferentially improved women’s accuracy [65].

Reference placement and focality refine—not replace—these principles. Several studies used extracephalic references (e.g., deltoid/arm) to reduce unintended cortical co-polarisation at the return electrode, yet still observed sex-dependent effects. In mind-wandering, cathodal mPFC stimulation with an extracephalic anode reduced mind-wandering in men but not women [45]. In moral judgment, a deltoid reference was also used, and women showed stronger polarity-differentiated choice changes than men [46]. In inequity aversion, rTPJ anodal stimulation with an arm reference produced a context-specific Treatment × Sex interaction (stronger equity shift in women under disadvantageous inequity) [69]. Other studies used cheek references and still reported sex-contingent changes, including attenuation of sham-condition gender differences in PPC mental rotation and sex-dependent pay-ratio sensitivity changes under TPJ stimulation [65,70]. Conversely, focal HD-tDCS often produced crisp interaction patterns consistent with sex-specific sensitivity of targeted hubs: a 4 × 1 ring montage over rDLPFC yielded robust sex × stimulation interactions in a fairness paradigm [44], and a concentric HD montage over dmPFC improved RMET accuracy in women while men showed a numerical decline, with regional specificity suggested by a matched comparison stimulation site [61]. At the same time, conventional montages can also show sex moderation when timing and outcome sensitivity align, indicating that focality is not the only route to detectable sex-by-stimulation effects [51]. A related mPFC study targeting implicit gender stereotyping provides a complementary pattern: using a conventional midline Fpz–Oz montage, anodal stimulation reduced men’s D-IAT scores relative to sham, whereas cathodal stimulation did not differ reliably from sham and women showed no significant stimulation-related change. Thus, mPFC stimulation did not simply shift social bias uniformly; its behavioural effect depended on participant gender and was selective to the implicit measure rather than the overall explicit sexism score [43].

Finally, two results link these montage/geometry observations to mechanism. First, an in silico study using a standard F3–Fp2 montage reported systematically lower modelled E-field intensities in males than females across regions (independent of Alzheimer’s status), reinforcing that identical settings can yield sex-linked differences in delivered cortical dose under some montages [54]. Second, an individualised-dosing experiment that scaled current to equalise modelled target current density found that sex associations observed under fixed dosing were attenuated or absent when dosing was individualised, suggesting that part of apparent sex moderation in behavioural response can reflect sex-linked differences in effective target engagement under one-size-fits-all dosing [55]. Overall, this body of evidence argues against treating “sex effects” as a fixed participant trait. Instead, sex moderation in tDCS is most consistently expressed as a contingent property of how and where stimulation is delivered—sex × montage × target (and often task/endpoint) interactions—consistent with the idea that montage-specific recruitment of neural and peripheral structures is shifted by sex-linked anatomy and related factors under otherwise identical device settings [24,25,26,28,33,36,37,39,42,44,45,46,54,55,64,65,69,70].

**Figure 5:**
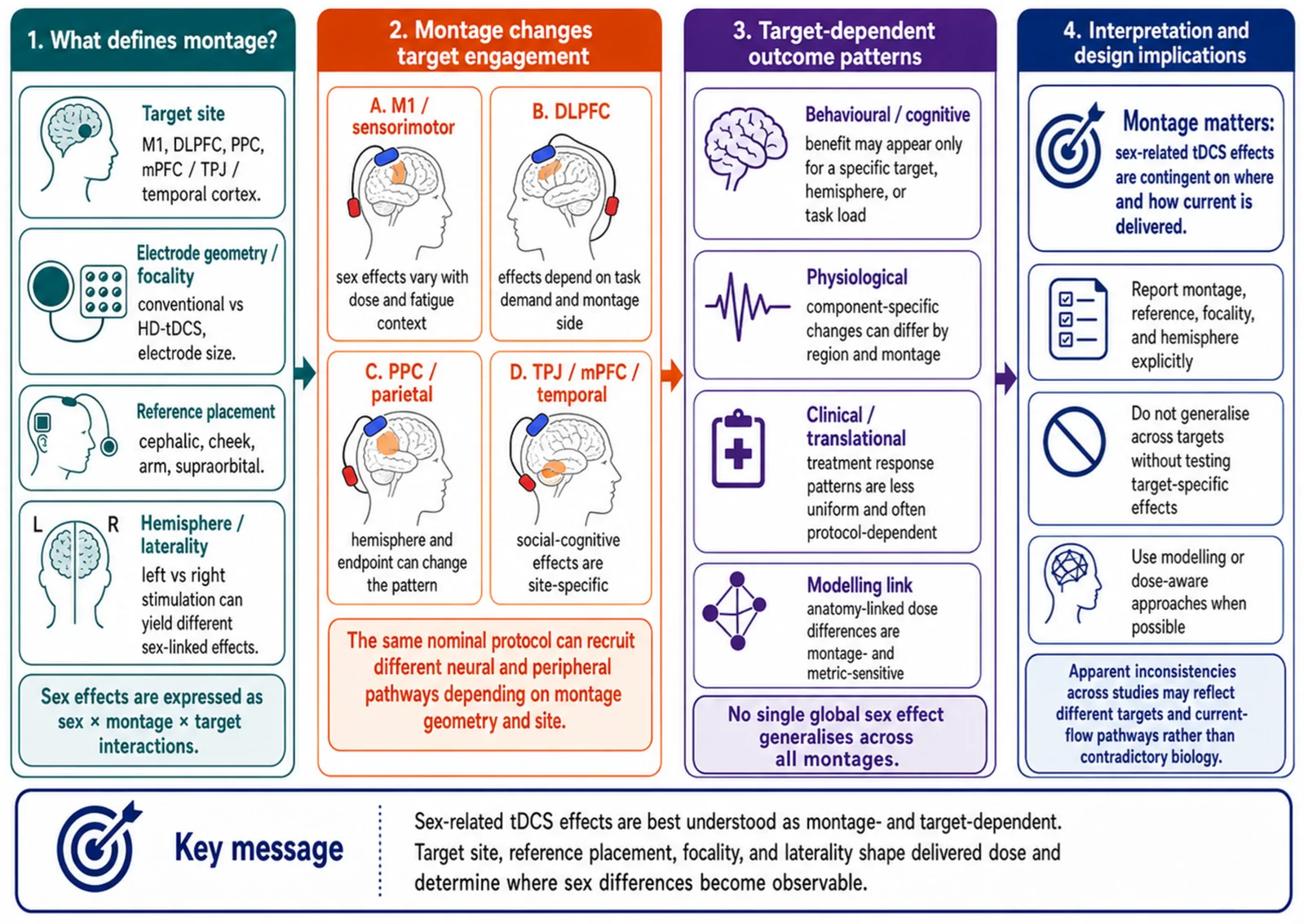
Montage and target dependence of sex-related tDCS effects. The figure illustrates that sex-related tDCS effects depend on how and where current is delivered. Target site, electrode geometry, focality, reference placement, and hemispheric laterality can alter target engagement and produce different neural, behavioural, or physiological response patterns. The same nominal stimulation protocol may therefore recruit different cortical and peripheral pathways depending on montage geometry and anatomical context. This supports the interpretation that apparent inconsistencies across studies may reflect target- and current-flow-dependent effects rather than contradictory biological findings.

The montage- and target-dependent nature of sex-related tDCS effects is summarised in Figure 5, which illustrates how target site, electrode geometry, reference placement, and laterality can shape delivered dose and produce different behavioural or physiological response patterns across studies.

### 3.4. Outcome Dynamics and Temporal Structure: Sex Effects Often Emerge in Trajectories, Late Phases, and Delayed After-Effects

Across behavioural and physiological studies, sex differences in tDCS response are often expressed in how performance changes unfold over time, rather than as simple baseline differences. Many paradigms show minimal or no sex differences at pretest. However, clear sex moderation frequently emerges in pre–post change, learning trajectories, error patterns, or late-phase performance once participants adapt their strategies or face higher control demands.

In executive control tasks, inhibition speed (SSRT) improved with practice regardless of sex or stimulation. Yet sex moderated how stimulation influenced practice-related changes in Go-trial accuracy, suggesting an effect on the adaptation or learning component rather than the stopping process itself [31]. In multi-session PASAT training, baseline performance did not differ by sex, but females showed larger overall gains.

Critically, the sex difference was polarity-specific: females benefited more than males under anodal stimulation during training, whereas cathodal and sham did not show the same sex differentiation in gains [35]. Similarly, in the Iowa Gambling Task, the sex-dependent tDCS signature was interactional and session-linked: women improved with anodal rOFC stimulation while men improved over time largely regardless of stimulation, making stimulation “matter” more for women in that design [42]. Working-memory findings reinforce that sex moderation can be conditional on task phase and difficulty: stimulation effects were absent at low-moderate loads but emerged at the highest load as a gender × tDCS interaction, with opposite ‘best montage’ patterns across sexes [39].

Several studies also indicate that sex moderation can concentrate in conflict-sensitive components or specific error structures rather than uniform performance shifts. In a sound–shape integration go/no-go task, reaction time effects on incongruent trials (active faster than sham) were not sex moderated, whereas the clearest sex-dependent effect appeared only for inhibitory control: NoGo accuracy showed a polarity × congruency × gender interaction driven by a male-specific decrement under cathodal stimulation in a congruent NoGo condition [40]. In affective evaluation, women rated facial emotions as more intense than men under sham, but anodal L-DLPFC stimulation largely reduced this gender divergence without parallel changes in implicit racial attitudes, illustrating that sex-linked differences can be selectively altered in one evaluative channel while leaving another unchanged [38]. The gender-stereotyping IAT study shows a similar component-specific structure. Men displayed a larger implicit stereotype effect than women, and anodal mPFC stimulation selectively reduced this male bias. Decomposition of the IAT suggested that the effect reflected a smaller congruent–incongruent performance gap, particularly in reaction-time and rate-correct-score measures, rather than a general speeding/slowing effect or a parallel change in explicit sexism. This makes [43] another example in which sex moderation appears in the conflict-sensitive structure of task performance rather than as a uniform behavioural shift. In parietal attention, women’s baseline manual line-bisection bias varied across menstrual-cycle phases. Yet stimulation did not reliably alter bisection; instead, stimulation-linked change was detectable on a different endpoint (computerised midpoint recognition) and primarily in men, underscoring that “where” sex effects appear can be endpoint-specific even within the same study [64]. In mental rotation, stimulation altered not only mean performance but also the presence of a sham-condition gender gap, particularly under right-PPC stimulation, indicating that sex moderation can involve eliminating or reshaping an existing performance divergence rather than amplifying it [65]. In social–economic judgment, right-TPJ stimulation did not produce a pay-ratio–independent shift in investment attractiveness; instead, it sharpened sensitivity to high versus medium pay-ratio disclosures in a way that concentrated in women (especially inexperienced women), again emphasising interaction structure and context dependence rather than uniform shifts [70].

The temporal structure, including online vs. quasi-offline delivery, delayed post-stimulation measurement, and late-task phases, further shapes detectability. In the visual cortex, sex differences were not apparent immediately after stimulation for either polarity. Later, after anodal stimulation, women showed greater facilitation than men at ~10 min post, while cathodal effects did not separate the sexes in the same way [51]. In prefrontal MRS, the principal Glx elevation was delayed (stronger in the second half of stimulation and post), whereas sex interactions appeared for GABA and NAA without a clear sex × stimulation × time interaction, suggesting that some sex effects reflect overall active–sham shifts while other stimulation-linked changes have delayed expression [50]. In decision-making, HD-tDCS effects on IGT outcomes were most evident in later, risk-dominant trials, consistent with the view that stimulation—and any sex moderation—may preferentially affect consolidation of strategy or exploitation under uncertainty rather than early exploration [52]. Across studies, timing is therefore not a simple binary determinant: sex moderation can arise with stimulation delivered during the cognitive process of interest (online) or assessed after stimulation (quasi-offline), but it tends to become most visible when the measured endpoint captures a stimulation-sensitive transition (e.g., late-stage learning, conflict engagement, risk-phase exploitation) [44,47,49,52,62].

Finally, even when sex does not significantly moderate an outcome, other contextual factors can gate stimulation effects in ways that matter for interpretation. In the Stop-Signal work, background acoustics significantly moderated the Stimulation × Practice effect on SSRT, whereas the corresponding higher-order interaction including sex was null, implying a general state/context dependence of inhibition plasticity under tDCS that can be independent of sex [31]. In fatiguability protocols, stimulation delivered during or just before the fatigue task produced sex moderation most clearly at higher intensity and, within women, depended on oestrogen state—illustrating that time course and physiological state can jointly determine whether stimulation-linked effects appear in behaviour or physiology [27,30]. Overall, these findings support a synthesis in which sex effects in tDCS are frequently dynamic: they emerge in trajectories, late phases, or delayed after-effects, and they often localise to specific components of performance or physiology rather than producing broad baseline shifts across all measures [31,35,38,39,40,42,50,51,52,64,65,70].

### 3.5. Sensation, Blinding, and Safety: When Sex Differences in Experience Can (and Cannot) Explain Outcome Moderation

Subjective experience during tDCS can influence outcomes by affecting blinding, expectancy, and task engagement. These factors are particularly relevant at higher intensities, where differences in perceived sensation become more pronounced. A tolerability-focused re-analysis found strong evidence that perceived sensations increase with intensity overall and that women rated sensations as more severe than men during active stimulation—especially at 4 mA, where the sex difference in severity was large [34]. Consistent with this, the leg fatiguability study that detected a sex × intensity interaction also documented intensity-linked challenges to blinding and detailed sensation profiles, indicating that higher intensities can shift subjective experience in ways that are not fully eliminated by standard sham methods [30]. Similarly, in the mPFC gender-stereotyping study, participants reported no post-stimulation scalp pain or headache, yet anodal stimulation selectively reduced implicit stereotyping in men, again arguing against a simple discomfort-based explanation for the observed sex × stimulation effect [43]. Together, these findings support the pragmatic inference that at high intensity, sex differences in discomfort and blinding integrity are plausible mediator pathways for sex differences in behavioural response [30,34].

Modelling work provides a mechanistic rationale for why experiential differences could be sex-linked. One simulation study reported males receiving higher modelled cortical current density than females under certain configurations and interpreted this as implying that, under comparable device settings, females might experience proportionally more scalp current and discomfort because less applied current reaches cortex and more is dissipated in superficial tissues [24]. Although the direction of modelled sex differences is not uniform across pipelines and montages (e.g., some modelling reports higher whole-brain EF in females, and large-cohort simulations show age-dependent reversals) [28,33], the general point remains robust: if sex-linked anatomy changes how current partitions between scalp/skull and brain, then sex differences in sensation, blinding, and expectancy become credible contributors to sex-by-stimulation effects on behaviour [24,28,33].

At the same time, converging evidence argues against a simple “sex moderation is just unblinding” account—particularly under standard (≈0–2 mA) dosing. A large Bayesian safety trial spanning children and adults and systematically varying intensity from 0 to 2 mA across diverse montages found moderate evidence for no meaningful male–female differences in side-effect intensity attributable to stimulation; the only statistically detectable sex difference occurred at a pre-stimulation baseline time point, consistent with a reporting difference rather than a stimulation-induced effect [60]. Several sham-controlled cognitive/social studies also reported low discomfort and acceptable blinding overall while still observing sex-dependent stimulation effects on specific outcomes—for example, intention attribution reaction time under mPFC stimulation [36], sex-dependent error-direction changes under bilateral temporal stimulation [37], and sex-contingent fairness- or pay-ratio–sensitivity effects under right-TPJ stimulation in large samples reporting comfortable procedures on average [70]. In the fairness HD-tDCS study, acceptability and perceived stimulation influence did not differ by sex, yet robust sex × stimulation interactions were observed in fairness-related behaviour, making a pure tolerability/demand-characteristics explanation less likely there [44]. Similarly, in mind-wandering work, discomfort ratings did not differ across gender-by-condition subgroups even though sex moderation was observed for the primary outcome in some analyses [45]. These patterns suggest that sex-by-stimulation effects can arise through neural mechanisms, task strategy, or subtler experiential differences that are not fully captured by simple blinding-guess or single-item discomfort measures—even when gross tolerability appears adequate [36,37,44,45,70].

Clinical and applied studies further reinforce this distinction: acceptable tolerability does not preclude sex-contingent efficacy patterns. In schizophrenia, participants completed a double-blind 10-session bi-frontal protocol with objective cognitive gains observed without sex moderation on primary endpoints, indicating that tolerability was sufficient and that sex differences were not automatically expressed in outcomes [67]. Conversely, open-label real-world depression work reported clinical improvement in both sexes, but without sham controls, expectancy and sensation pathways cannot be ruled out as contributors to any subgroup differences [68]. Overall, the most defensible synthesis is conditional: at higher intensities, sex differences in sensation and blinding challenges are empirically supported and can plausibly mediate behavioural differences; under standard parameters, the best evidence suggests minimal sex differences in stimulation-induced side effects, and repeated demonstrations of sex × stimulation effects in well-tolerated, sham-controlled designs indicate that experiential pathways are not a sufficient general explanation for sex moderation across the literature [30,34,44,60,70].

### 3.6. Task Difficulty, Conflict, and Adaptive Demand as Amplifiers of Sex Moderation

Sex differences in tDCS outcomes are most consistently observed under high task demands. Examples include high working-memory load, strong stimulus–response conflict, or performance contexts that require sustained adaptation and strategy updating. This “difficulty amplification” pattern helps reconcile two common findings: many studies show weak or null sex main effects on simpler endpoints, yet reveal clear sex × stimulation interactions once the paradigm stresses executive resources or pushes participants into late-phase learning and exploitation regimes.

A clear example comes from verbal working memory under interference, where demand was manipulated by increasing load and adding inhibitory pressure. Sex moderation did not appear at lower loads; instead, it emerged specifically at the highest load as a WM load × gender × tDCS interaction. Critically, the direction of benefit depended on montage: men showed the clearest improvement relative to sham under left DLPFC stimulation at the highest load, whereas women showed a relative advantage for right DLPFC stimulation compared with left stimulation at the same high-load level—illustrating that under maximal control demands, where stimulation helps can diverge by sex [39]. Related evidence comes from tasks where “difficulty” is operationalised as conflict rather than memory load. In a multi-sensory sound–shape matching go/no-go task, the dominant signature was a large congruency cost, and sex-linked differences were concentrated where conflict was highest: women were less accurate than men on incongruent Go trials, while no sex difference emerged on congruent Go trials. Stimulation did not broadly change Go accuracy, but it did modulate conflict-sensitive components: reaction times on incongruent trials were faster under active stimulation than sham, and inhibitory control (NoGo accuracy) showed a polarity × congruency × gender interaction driven by a male-specific decrement in a cathodal/congruent NoGo condition [40]. Stimulation produced minimal effects when stimulus–response mappings were straightforward. In contrast, sex-related differences emerged under conflict conditions that required participants to inhibit a prepotent response. This pattern suggests that sex may moderate tDCS effects most clearly when executive-control demands are high. The gender-IAT study extends this conflict-amplification pattern to implicit social stereotyping. Both men and women were slower in incongruent than congruent blocks, but men showed a much larger implicit stereotype effect overall. Anodal mPFC stimulation reduced men’s D-IAT scores and narrowed the congruent–incongruent RT/RCS gap, whereas women showed no comparable stimulation effect. This suggests that sex moderation can emerge when stimulation targets neural systems involved in regulating stereotype-congruent versus stereotype-incongruent response conflict, even when explicit attitude scores remain largely unchanged [43].

Difficulty can also amplify sex moderation through learning dynamics rather than single-session accuracy. In the Stop-Signal Task, stimulation did not alter the canonical stopping metric (SSRT) in a sex-dependent way, but sex moderated how stimulation shaped practice-related change in Go-trial accuracy—indicating that sex moderation can emerge in adaptive trajectories under repeated high-attention performance rather than in a single inhibitory endpoint [31]. Similarly, during PASAT cognitive control training, sex differences were absent at baseline but emerged in training gains, and those gains were polarity-specific: females showed a clearer anodal-associated advantage during training, whereas males did not show the same stimulation-linked improvement relative to sham [35]. These findings indicate that sex-by-stimulation interactions are most likely to emerge in demanding and adaptive contexts. Such contexts engage fatigue resistance, frustration tolerance, and strategy selection.

Parietal and social–economic paradigms provide converging examples in which the “sex effect” is interaction-structured and becomes visible under higher uncertainty, conflict, or gap-relevant performance contexts. In mental rotation, stimulation effects depended on stimulus type and angle, and the stimulation × gender interaction indicated that gender differences present under sham were attenuated under PPC stimulation—particularly right PPC—rather than being uniformly amplified [65]. In inequity aversion, right-TPJ stimulation selectively increased equity choices under disadvantageous inequity, but not advantageous inequity, and this context-specific effect was additionally moderated by sex (stronger fairness shift in women) [69]. In investment judgments, stimulation did not uniformly shift attractiveness ratings; it sharpened sensitivity to high (vs medium) pay-ratio disclosures, with the interaction concentrating in women—especially those without investment experience—suggesting that baseline uncertainty/experience functions like a “difficulty amplifier” for whether sex moderation becomes behaviourally expressible [70].

Taken together, these studies support a practical interpretation: sex moderation in tDCS is often conditional—not “women respond more” or “men respond more,” but sex differences emerge when tasks force high-load or high-conflict control states, and the direction of benefit can depend on montage, target hemisphere, and the specific control component being measured [31,35,39,40,65,69,70]. This also implies that null sex effects in low-demand tasks should not be treated as strong evidence against sex moderation; they may simply indicate that the paradigm did not sufficiently stress the cognitive operations where stimulation-linked sex differences are most likely to manifest behaviourally.

**Figure 6:**
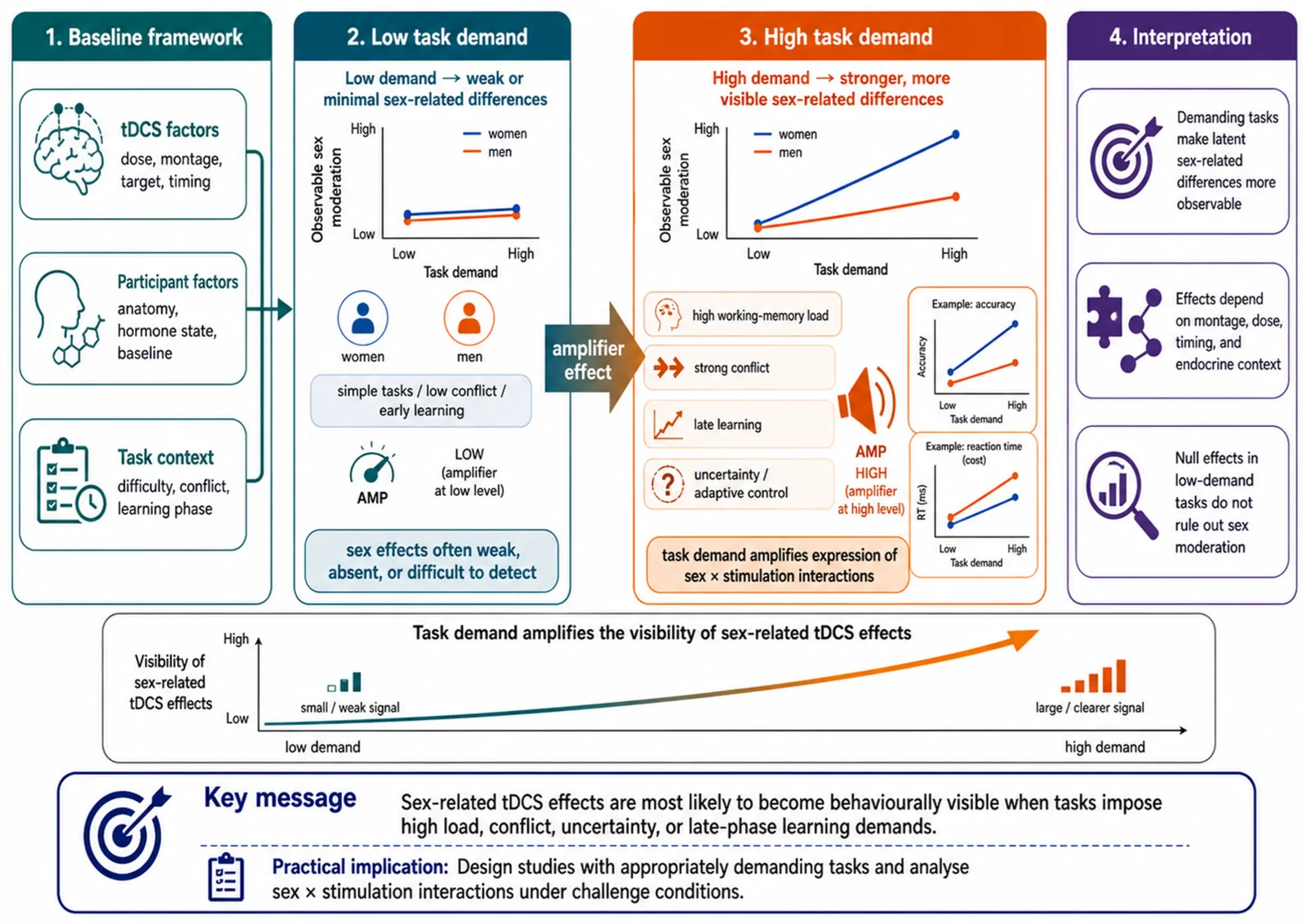
Task-demand amplifier model of sex-related tDCS effects. The figure proposes that task demand can amplify the behavioural visibility of sex-related stimulation effects. Under low-demand conditions, sex × stimulation interactions may be weak, absent, or difficult to detect. Under high working-memory load, strong conflict, uncertainty, adaptive control demands, or late learning phases, latent sex-related differences in stimulation response may become more observable. The model emphasises that null findings in simple or low-demand tasks do not necessarily rule out sex moderation, because task context may determine whether stimulation-induced differences become behaviourally expressed.

The role of task demand as an amplifier of sex-related stimulation effects is presented in Figure 6, which illustrates how low-demand tasks may produce weak or undetectable sex moderation, whereas high-load, high-conflict, uncertain, or late-learning conditions can make sex × stimulation interactions more behaviourally visible.

### 3.7. Hormonal and Endocrine State as a Key Parameter That Can Masquerade as or Magnify “Sex” Effects

Across this literature, some apparent sex differences in tDCS response may reflect fluctuating endocrine state within women rather than stable female–male differences. However, the strength of this interpretation depends on how hormonal status was established. Direct measurement of circulating hormones provides stronger evidence than classification based only on menstrual-cycle phase, because estradiol and progesterone covary across the cycle and exhibit substantial interindividual and inter-cycle variability. 

Two studies provide relatively direct evidence that endocrine state can modify stimulation outcomes, although even these findings should not be interpreted as demonstrating an isolated effect of estradiol. In the leg-fatiguability study, estradiol was assessed across sessions, and 4 mA anodal M1 stimulation increased extensor fatiguability primarily during sessions with higher estradiol levels [27]. In the DLPFC TMS–EEG study, blood assays were used to verify lower- and higher-estradiol conditions, and enhancement of the P200 response after tDCS was clearest in the higher-estradiol group [32]. The latter study therefore supports an association between measured estradiol status and tDCS-induced cortical responses. Nevertheless, if progesterone was not measured concurrently, neither study can fully separate estradiol-specific effects from the broader endocrine context, including progesterone concentration, the estradiol-to-progesterone ratio, ovulatory status, or other cycle-related physiological changes. These findings are therefore best described as evidence of endocrine-state-dependent responsiveness, with estradiol as a measured correlate rather than an established sole causal mechanism.

Other studies addressed hormonal variability through study design rather than direct endocrine measurement. One emotion-rating study tested women within a specified follicular window to reduce cycle-related variability [38]. A PASAT training study recorded menstrual status and hormonal-contraceptive use and reported that these variables were similarly distributed across stimulation groups [35]. Such procedures reduce the likelihood of gross imbalance between experimental conditions, but they do not establish participants’ circulating estradiol or progesterone concentrations. Calendar-based or self-reported cycle phase is an imperfect proxy for endocrine state because cycle length, ovulatory timing, and hormone concentrations vary both between and within individuals. These studies should therefore be interpreted as controlling approximately for cycle timing, not as testing hormone-specific moderation.

A further study tested naturally cycling women during menstrual, follicular, and mid-luteal phases, with phase classification supported by ultrasonographic markers [64]. Women showed phase-related variation in baseline manual line-bisection bias, but left-parietal anodal stimulation did not reliably alter bisection performance in any phase. Under the same montage, men showed improved accuracy on a computerised midpoint-recognition task, whereas women showed no comparable stimulation-related benefit. Because circulating estradiol and progesterone were not reported as direct continuous predictors, these findings demonstrate variation associated with menstrual-cycle phase rather than an isolated effect of either hormone. In particular, the luteal phase should not be described simply as a low-oestrogen condition, because it is characterised by substantial progesterone exposure and variable estradiol concentrations.

Mechanistically, these patterns align with a two-source view of “sex effects” in tDCS: (i) anatomy-linked differences in delivered intracranial dose under identical device settings (as shown in multiple modelling pipelines and montages) [24,25,28,33,42], and (ii) state-dependent neurophysiology linked to hormones that can modulate excitability and plasticity during or after stimulation [27,32], with cycle-related controls in [38]). In practice, studies that treat sex as a binary covariate without measuring hormonal state may conflate stable sex-linked factors with fluctuating endocrine context—potentially explaining why sex moderation can be selective, variable across tasks, and sometimes inconsistent across replications.

**Figure 7:**
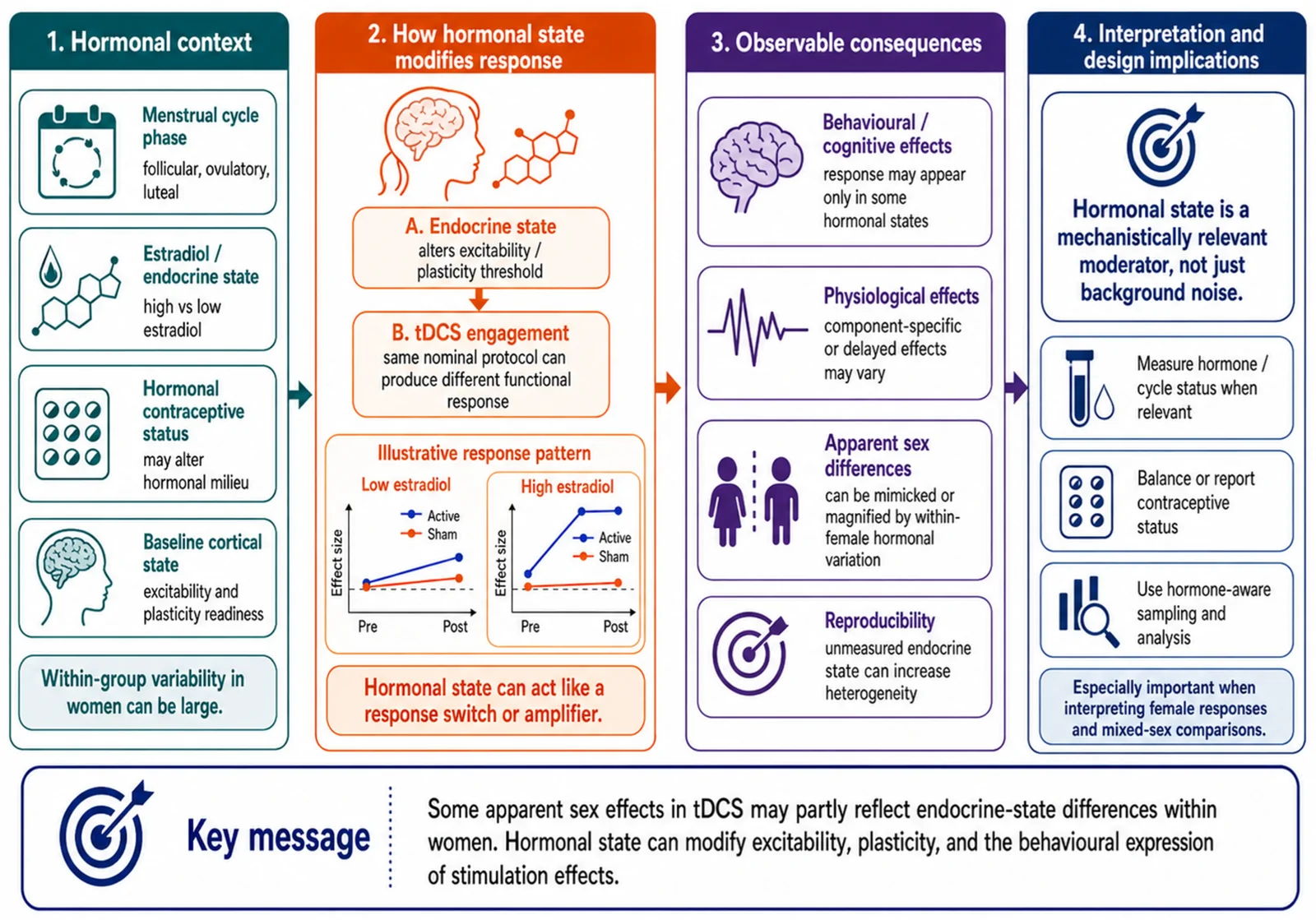
Hormonal state as a modifier of tDCS responsiveness. The figure summarises how menstrual-cycle phase, estradiol level, hormonal contraceptive status, and baseline cortical state may influence tDCS-induced effects. Hormonal state may alter excitability thresholds, plasticity readiness, and the behavioural or physiological expression of stimulation effects. Consequently, apparent sex differences in mixed-sex studies may partly reflect unmeasured endocrine variation within women. The figure highlights the need to measure, balance, or report hormonal status when relevant, particularly in studies examining female responses or mixed-sex comparisons.

The possible role of hormonal state as a modifier of tDCS responsiveness is summarised in Figure 7, which shows how menstrual-cycle phase, estradiol level, hormonal contraceptive status, and baseline cortical excitability may alter stimulation-induced behavioural and physiological outcomes.

### 3.8. Participant Parameters and Clinical Context: Age, Baseline Ability, and Biological Modifiers Can Outweigh Sex as Standalone Predictors

Across both basic and clinical/translational studies, sex moderation rarely acts in isolation. Instead, age, baseline ability, clinical status, and biological modifiers frequently shape whether sex differences in tDCS response are detectable and how they should be interpreted. This is clearest in datasets where sex effects only emerge within specific age–performance strata. In a re-analysis of anodal mPFC ToM stimulation, pronounced active-minus-sham slowing in ToM reaction time was observed primarily in low-performing older women, while high-performing older women and younger participants showed minimal or no sensitivity; here, the apparent sex effect is inseparable from an age × baseline-performance interaction [49]. Similarly, in an older-adult intervention combining cognitive training with stimulation, sex alone did not significantly moderate training gains in the primary models, although exploratory decompositions suggested different “where benefits appear” patterns under an age × sex × stimulation trend (e.g., stimulation-type effects differing across age and sex strata) [63]. These results highlight a general principle: what looks like sex moderation can reflect differential headroom, strategy, or neurobiological reserve that is unevenly distributed across sex-by-age subgroups rather than a direct sex-linked mechanism.

Clinical ageing contexts provide additional examples in which sex interacts with biological modifiers beyond demographics. In MCI patients receiving a two-week home-based left DLPFC course, sex significantly moderated changes in DMN–SN connectivity (females showing increased coupling compared to males), and sex also participated in a higher-order interaction with BDNF genotype for salience-network intra-connectivity—indicating that sex may function as one node in a broader moderator network rather than as a dominant standalone stratifier [59]. Such findings are consistent with the view that in clinical or ageing populations, heterogeneity in neurodegeneration, genetic plasticity factors, and network integrity can gate stimulation effects, and sex may matter most when it aligns with these other determinants.

Clinical studies further indicate that sex may be less predictive than other participant characteristics, including age, education, baseline severity, and disease subtype. These clinical and translational findings are discussed in detail in Section 3.9 [66,67,68].

Non-clinical studies also illustrate how baseline “experience” or ability can function like a participant-parameter moderator that interacts with sex. In investment evaluation, right-TPJ stimulation primarily altered sensitivity to high pay-ratio disclosures in women, especially those without investing experience, implying that baseline knowledge/strategy can gate whether fairness cues influence judgment under stimulation [70]. More broadly, across cognitive and social paradigms, baseline performance differences can shape headroom for change and thereby the visibility of sex-by-stimulation interactions, reinforcing that “sex effects” often reflect higher-order moderation structures rather than simple male–female contrasts.

Finally, when hormonal or cycle-related controls are present, they constrain interpretation by reducing within-female variability that can otherwise masquerade as sex effects. The prefrontal MRS study scheduled female participants outside menstruation, a design choice that may reduce cycle-phase variance in metabolites such as GABA and thus influence detectability of sex-by-stimulation interactions [50]. Conversely, many behavioural studies did not measure hormones or tightly control cycle timing, leaving open whether part of apparent sex moderation reflects fluctuating endocrine state within women or correlated covariates (trait differences, baseline strategies, symptom profiles) rather than stable sex-linked mechanisms [44,46,47,48,57,58,61,62].

Overall, the evidence supports treating sex as a context-dependent moderator embedded in a larger system of participant parameters. Sex moderation can be pronounced in specific age–ability strata, can interact with genotype and network integrity in clinical ageing, and can disappear once other key covariates (age/education) are modelled—making “sex” a potentially useful stratifier in some settings but an incomplete explanation without considering age, baseline performance, experience, and biological modifiers [49,59,63,66,67,70].

### 3.9. Clinical and Translational Contexts: Sex Can Moderate Trajectories and Network Mechanisms, but Often Alongside Other Moderators

Clinical and translational studies extend “sex effects” beyond modelled dose and lab-task performance by focusing on symptom trajectories, therapy-coupled outcomes, and network-level mechanisms. In these settings, sex can meaningfully stratify response patterns, but interpretation is often constrained by non-randomised care pathways, baseline severity differences, and the presence of strong co-moderators such as age, education, or disease subtype.

In a large tinnitus clinic analysis that included an HD-tDCS arm, sex moderated improvement trajectories differently depending on treatment type, producing a significant time × sex × therapy interaction: women improved more than men in the HD-tDCS arm and in orofacial therapy, whereas the pattern reversed in TRT + CBT (men improved markedly; women did not), and TRT + EMDR showed no sex difference in improvement [29]. Because treatment assignment was not randomised and baseline differences (including higher baseline TFI in women in some groups) were present, the most defensible interpretation is that sex is a meaningful stratifier of real-world outcomes and pathway-dependent response, but not necessarily evidence that tDCS alone caused the sex differences in that clinic setting [29]. This study therefore illustrates a broader translational point: in routine-care contexts, “sex moderation” can reflect how sex correlates with baseline severity, comorbidity profiles, and treatment allocation as much as differential neurophysiological responsiveness.

Mechanistic clinical analyses provide more direct evidence that sex can shape stimulation-linked neural response in domain-specific ways. In PPA therapy paired with tDCS, resting-state fMRI analysed via CAP regression showed sex-dependent network effects: a DMN-like subnetwork exhibited a significant active-minus-sham effect in men but not women, while a language-network component showed the opposite pattern (significant in women but not men), even after adjusting for age, variant, and language severity [41]. This is a particularly clear demonstration that sex moderation can differ by network domain within the same protocol and dataset, reinforcing the broader theme that sex effects are often outcome- and system-specific rather than global.

At the same time, newer clinical moderator analyses underscore that sex is not always dominant once other patient parameters are modelled. In subacute post-stroke aphasia therapy paired with individualised left-hemisphere anodal tDCS, age and education significantly moderated the added benefit of tDCS on short-term naming change, whereas sex did not (sex × treatment interaction n.s.), suggesting that cognitive-reserve proxies and recovery stage can outweigh sex in explaining response heterogeneity in early stroke recovery [66]. In schizophrenia, a double-blind 10-session bifrontal protocol improved Working Memory and Neurocognition versus sham without sex moderation of objective gains, though sex differences emerged in how subjective “felt improvement” aligned with objective change—implying that sex may influence appraisal–performance coupling even when it does not alter primary efficacy [67]. An open-label depression course delivered with a commercial bifrontal device also reported clinical improvement in both sexes with numerically larger effects in males within a moderate/severe subgroup, but the lack of sham control and small sex-stratified samples limit causal inference about sex differences in tDCS efficacy there [68].

Taken together, clinical and translational evidence supports a conditional synthesis: sex can moderate symptom trajectories and stimulation-linked network mechanisms (and may do so differently across network domains), but in many patient populations sex competes with—or is nested within—other determinants of heterogeneity such as baseline severity, age, education, disease subtype, and trial design constraints [29,41,66,67,68].

### 3.10. Outcome Quantification and Metric Choice: Where Sex Effects Appear Depends on What Is Measured

Across studies, “sex effects” are often not properties of raw task scores alone, but of how outcomes are quantified—change scores versus absolute performance, composite indices versus single items, computational parameters versus behavioural summaries, and physiological components versus global averages. This measurement heterogeneity matters because the same dataset can yield weak sex differences on one metric and clear sex × stimulation interactions on another, shaping synthesis and interpretability.

In several paradigms, sex moderation was visible primarily in interaction terms on change outcomes rather than in baseline levels. For example, mind-wandering work emphasised stimulation × gender effects on Δ scores (with men driving the principal mPFC result), illustrating that sex moderation may be expressed in pre–post shifts even when baseline group means, or global discomfort measures, do not differ markedly [45]. Conversely, some studies show baseline behavioural tendencies that create different headroom for stimulation and then interact with sex: in a deception task, men were more honest than women under sham, and right-anodal/left-cathodal stimulation selectively reduced deception among women, effectively eliminating a baseline gender gap; here, the “sex effect” is partly a baseline-strategy difference that conditions how stimulation changes behaviour [48]. These cases emphasise that sex moderation can reflect either differential susceptibility to stimulation or differential baseline policy/strategy that is preferentially altered by stimulation, and the distinction often depends on whether the analysis foregrounds baseline vs. change.

Computational or parameter-level outcomes can also tell a different story from aggregate performance indices. In Iowa Gambling Task modelling, stimulation effects were partly dissociable across behavioural net score versus PVL parameters: OFC stimulation altered the PVL shape parameter aaa without producing the same net-score benefit as DLPFC stimulation, and the reported sex interaction was strongest for the behavioural net-score outcome rather than being framed as a sex-specific computational shift [52]. In mental rotation, stimulation effects were decomposed into chronometric regression intercepts and slopes; stimulation-linked reductions in reaction time (especially under right PPC) corresponded to changes in baseline processing components rather than only rotation-rate changes, while sex differences were evaluated at both mean-performance and parameter levels [65]. These examples illustrate that apparent sex moderation can be “located” in different computational components depending on whether one analyses totals, learning-phase segments, or parameter estimates.

Composite construction and multi-measure aggregation can further change where sex effects emerge. In investment evaluation, overall perceived investment potential was a composite, but the stimulation-by-pay-ratio effect localised most strongly to a specific subcomponent (“expected stock price appreciation”) in women; exploratory mediation tests then linked stimulation to perceived risk and downstream earnings expectations in inexperienced women, highlighting how disaggregating a composite can reveal the pathway through which sex moderation expresses itself [70]. In frustration-induced aggression, three behavioural indices were moderately intercorrelated and formed a reliable composite; the stimulation × gender interaction was expressed most clearly at the composite level as a disappearance of the typical male > female aggression gap under active stimulation [62]. By contrast, in dlPFC aggression paradigms that separate proactive and reactive components, sex moderation depended on subtype and montage direction, producing patterns where the same stimulation increased aggression in some male contexts while reducing reactive aggression in females under a different montage—showing that aggregation can either clarify (when indices tap the same latent construct) or obscure (when subcomponents diverge) [57,58].

Clinical and translational work provides parallel examples where sex differences can appear in mapping between subjective and objective outcomes rather than in objective change itself. In schizophrenia, sex did not moderate objective MCCB change scores, yet sex differences emerged in the correspondence between subjective “felt improvement” and objective gains (agreement metrics differing by sex for Neurocognition), implying that sex moderation can be present in metacognitive calibration or reporting alignment even when primary performance endpoints show no sex interaction [67]. This underscores that “sex effects” can reside in different layers—objective cognition, subjective appraisal, or their coupling—depending on outcome definition.

Overall, this evidence argues that synthesis should treat “sex effects” as metric-contingent: they may appear in change trajectories rather than baselines, in specific error or conflict components rather than global accuracy, in computational parameters rather than net scores (or vice versa), and in composite constructs only when aggregation matches the latent structure of the behaviour [45,48,52,57,58,62,65,67,70]. Consequently, across-study comparisons should prioritise aligning what is being compared (raw vs. change, early vs. late phase, composite vs. subcomponent, parameter vs. score) before interpreting heterogeneity as evidence for or against sex moderation.

**Figure 8:**
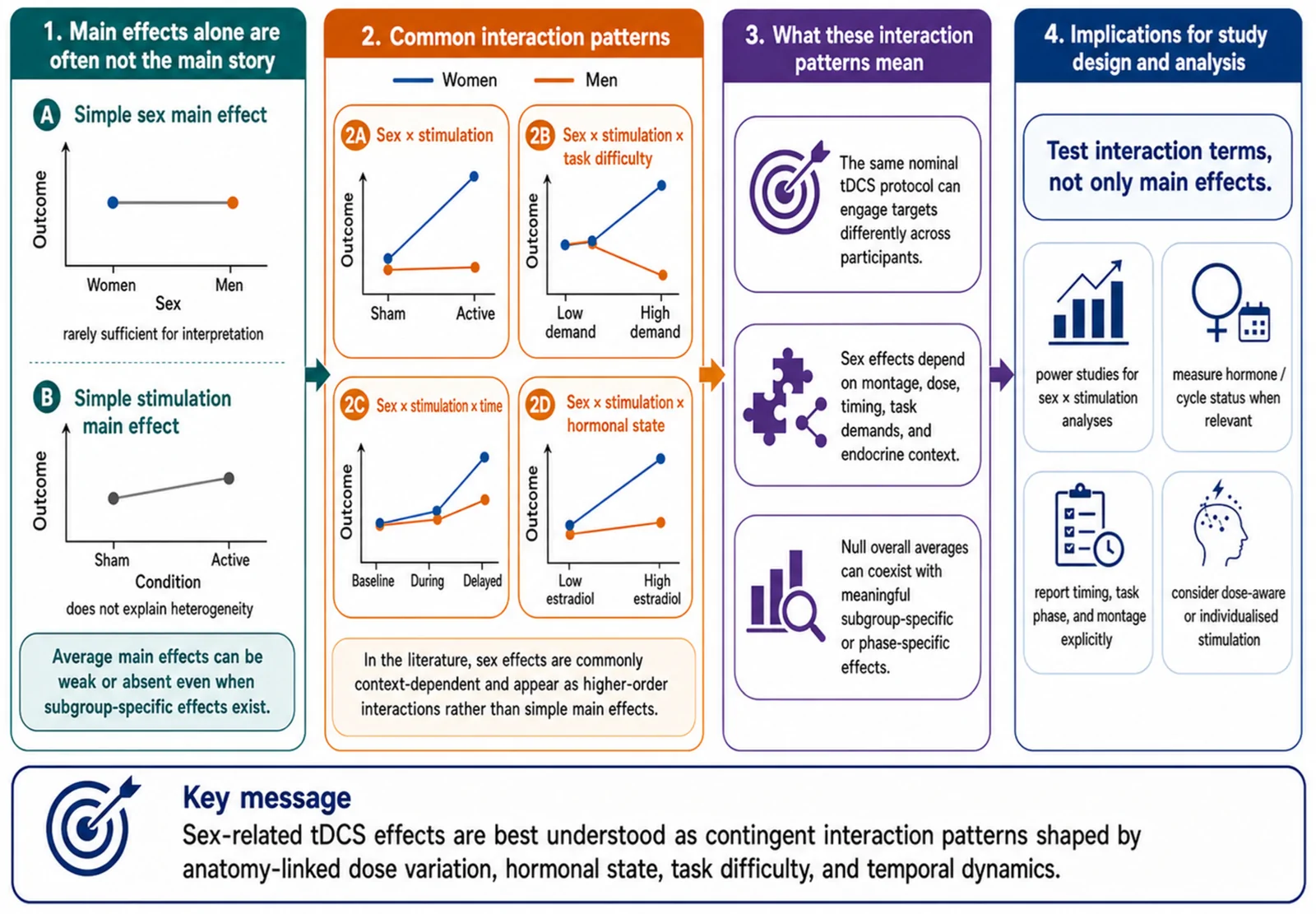
Sex-related tDCS effects as interaction patterns rather than simple main effects. The figure illustrates why male–female comparisons or stimulation main effects alone may be insufficient to explain tDCS heterogeneity. Sex-related effects may emerge as sex × stimulation, sex × task difficulty, sex × time, sex × hormonal state, or higher-order interaction patterns. Null average effects can therefore coexist with meaningful subgroup-specific, phase-specific, or context-specific effects. This framework supports analysing sex as a mechanistically relevant moderator rather than treating it only as a nuisance covariate.

The interaction-based nature of sex-related tDCS effects is illustrated in Figure 8, which shows why simple sex main effects or stimulation main effects may be insufficient and why sex-related effects often emerge as sex × stimulation, sex × task demand, sex × time, or sex × hormonal-state interactions.

### 3.11. Targeted Cortical System, Behavioural Domain, and Polarity: Sex Moderation Often Appears as Directionally Opposite, Context-Specific Effects in Association Networks

Across studies, sex-contingent tDCS effects are most consistently observed when stimulation targets association cortices involved in valuation, executive control, and social cognition. This is also true when outcomes require integrating competing motives or resolving conflict rather than producing simple speeded responses. Many of these sex effects are directional (women and men shift in opposite directions) and polarity- or context-dependent, rather than reflecting a uniform “women respond more” or “men respond more” pattern.

In social–economic and moral/valuation paradigms, sex moderation frequently takes the form of opposite-direction changes under the same stimulation. In a fairness task targeting rDLPFC with a focal HD montage, anodal stimulation tended to reduce women’s voluntary fairness but increase men’s, while simultaneously enhancing women’s sanction-induced compliance and reducing (or not improving) men’s incremental sanction response—yielding robust sex × stimulation interactions even when some within-sex contrasts were modest [44]. In a moral-judgment design targeting ventral prefrontal cortex (VPC) with an extracephalic reference, women’s utilitarian choices increased after anodal stimulation and trended downward after cathodal stimulation, whereas men’s utilitarian proportions were comparatively stable, indicating that polarity primarily differentiated female response profiles [46]. Theory-of-mind work targeting dmPFC similarly showed polarity-asymmetry in sex moderation: anodal stimulation improved RMET accuracy in women while men showed a numerical decline, producing a clear stimulation × sex interaction concentrated in the anodal condition [61]. Together, these studies illustrate a recurring theme in association hubs: sex moderation often concerns which direction the system is pushed under polarity-specific modulation, particularly for value-laden judgments and social inference [44,46,61].

This pattern is not confined to “social” tasks. Decision-making under uncertainty and learning-based choice also show sex-contingent response structures. In a sequential search paradigm, right-anodal/left-cathodal DLPFC stimulation improved accepted values and income most clearly in women, while men showed weaker within-condition shifts [47]. In HD-tDCS work on Iowa Gambling Task behaviour, stimulation condition interacted with sex for net score, and the clearest group-level stimulation effect was expressed differently in male versus female stratified analyses—again consistent with sex moderation emerging in strategy formation or late-phase exploitation rather than in generic task engagement [52]. Aggression studies targeting dlPFC and VLPFC further reinforce domain specificity: sex moderation depends on aggression subtype (proactive vs. reactive) and eliciting context (e.g., frustration-driven retaliation), producing cases where the same stimulation can increase aggression in some male contexts while reducing reactive aggression in females under a different montage or polarity configuration [57,58,62]. These findings converge on the idea that sex moderation becomes most visible when the stimulated network must regulate affectively salient impulses or uncertainty-driven strategy shifts rather than execute low-level motor output [47,52,57,58,62].

Polarity effects and directionality also appear in neurophysiological and metabolic endpoints, sometimes with asymmetries favouring inhibitory (cathodal-like) effects. In a retrospective motor-cortex analysis, women showed stronger and longer-lasting inhibitory after-effects following cathodal stimulation, with significant sex differences at multiple post-stimulation time points, while anodal after-effects did not show reliable male–female separation despite time-course nuances [53]. In prefrontal MRS, stimulation × sex interactions were detected for GABA and NAA (driven by females showing lower values in active vs. sham), whereas Glx increases were not sex dependent in the primary models. This suggests that sex moderation may sometimes be more readily expressed in inhibitory/metabolic markers than in excitatory composite signals [50]. Not all polarity-focused paradigms yield sex moderation, however: in a motor-learning crossover comparing anodal tDCS, hf-tRNS, and sham, performance improved over time largely irrespective of stimulation and showed no significant sex interactions in primary models [56]. This mix of outcomes indicates that polarity-by-sex effects are not guaranteed. They are conditional on target system, endpoint sensitivity, and task demands [50,53,56].

Even within anodal-only protocols, directionality can still be sex contingent and site-specific. Left-parietal anodal stimulation improved computerised spatial-attention accuracy in men but not women across menstrual phases [64]. In mental rotation, left-PPC anodal stimulation preferentially improved women’s accuracy, whereas right-PPC anodal stimulation more consistently improved accuracy/speed and reduced or eliminated the sham-condition gender gap [65]. Right-TPJ anodal stimulation increased equity choices primarily under disadvantageous inequity with a stronger shift in women [69] and sharpened sensitivity to high pay ratios in investment judgments in a way that concentrated in women, especially those without investing experience [70]. These results highlight that “direction” can mean different things across domains: opposite-direction within the same measure (e.g., fairness shifts), attenuation of a sham-condition sex gap (mental rotation), or sex-specific emergence of context sensitivity (TPJ fairness/pay-ratio effects) [44,65,69,70].

Overall, combining domain targeting with polarity effects yields a coherent synthesis. Sex moderation is most reliably expressed when stimulation engages association networks supporting valuation, control, and social inference. It also often appears as interaction-structured, directionally divergent effects that depend on polarity, context, and the specific control component being measured. This is opposed to stable, global sex main effects across tasks or targets [44,46,47,50,52,53,57,58,61,62,64,65,69,70].

**Figure 9:**
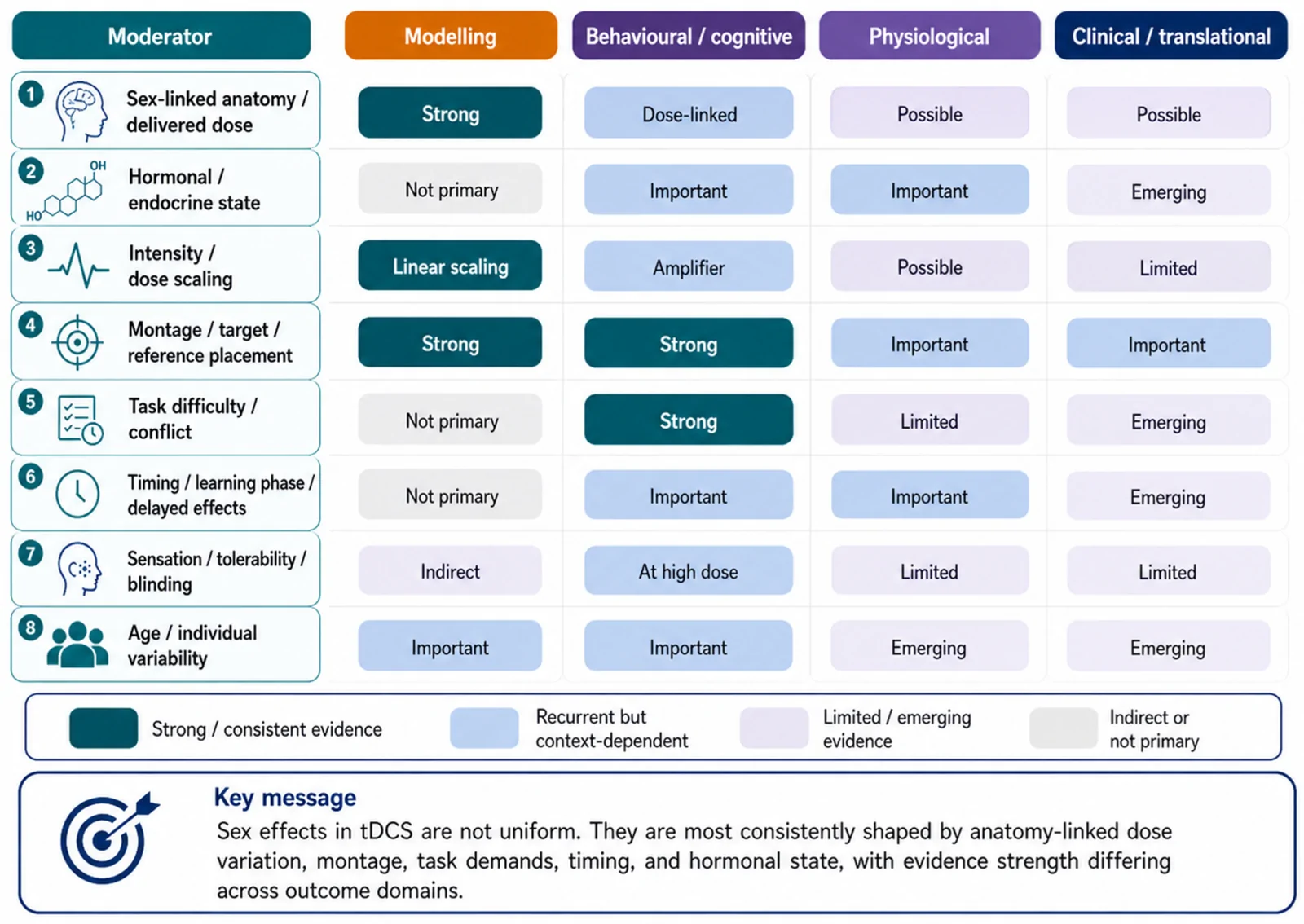
Matrix of moderators shaping sex-related tDCS effects across outcome domains. The figure summarises key moderators identified across modelling, behavioural/cognitive, physiological, and clinical/translational studies. Sex-related tDCS effects appear to be shaped most consistently by anatomy-linked dose variation, montage and target selection, task difficulty, stimulation timing, hormonal/endocrine state, intensity, sensation/tolerability, age, and individual variability. The matrix highlights that evidence strength differs across domains and that sex effects should be interpreted as context-dependent moderator patterns rather than uniform effects across all protocols or outcomes.

A cross-domain synthesis of the main moderators identified in this review is provided in Figure 9, which organises the evidence according to sex-linked anatomy and delivered dose, hormonal/endocrine state, stimulation intensity, montage and target, task difficulty, timing, sensation/tolerability, age, and individual variability.

## 4. Discussion

Gender differences between women and men are evident. Analysis of numerous studies has shown that women and men respond differently to tDCS. These differences are evident at the neurobiological, physiological, and behavioural levels. These differences help explain a common puzzle in tDCS research: that stimulation sometimes works better, sometimes worse, and sometimes not at all. This is an important piece of the puzzle explaining the null effects found in primary studies and reviews. This review is the first to focus on these gender differences and attempt to explain the mechanisms underlying these differences. This is not straightforward, as this is an under-explored area of research. Possible gender-related mechanisms responsible for differences in tDCS outcomes are discussed below.

### 4.1. Integrating Excitability Mechanisms with tDCS Effects

The common shorthand that “anodal stimulation excites and cathodal stimulation inhibits” is insufficient to explain sex-related variability. tDCS modifies an ongoing balance among excitation, inhibition, and plasticity. The magnitude, and sometimes the direction, of this effect depends on the physiological state of the cortex when stimulation is delivered. Sex hormones are a major source of that baseline-state variability because they modulate glutamatergic/NMDA-dependent excitability, GABAergic inhibition, and plasticity-enabling factors such as BDNF. The Rivas-Grajales review provides a clear scaffold for this: across the TMS literature, higher oestrogen and testosterone tend to be associated with greater cortical excitability, whereas higher progesterone is generally associated with reduced excitability and stronger inhibitory tone. Mechanistically, oestrogen is understood to increase excitability by enhancing glutamatergic neurotransmission and attenuating GABAergic responses, while progesterone (via neuroactive steroid actions, including allopregnanolone) facilitates GABA_A receptor–mediated inhibition and elevates inhibitory tone. These hormone-linked shifts are not abstract; they are captured in TMS-derived indices that map directly onto excitatory–inhibitory (E:I) balance. In naturally cycling women, high-oestrogen states are repeatedly associated with greater intracortical facilitation and larger motor-evoked responses, such as increased ICF during the late follicular phase and increases in MEP size around ovulation, whereas high-progesterone luteal states tend to show stronger inhibition, such as increased SICI and reductions in facilitation relative to follicular phases. Testosterone likewise trends in the “excitatory/plasticity-permissive” direction: in men, higher testosterone (and estradiol) has been associated with reductions in motor threshold, and in mixed samples higher testosterone has correlated with stronger PAS-like plasticity [71].

This matters for tDCS because the same nominal stimulation dose is effectively applied to different physiological substrates when baseline excitability differs. A cortex biased toward relatively greater excitation and plasticity—for example, during an endocrine state characterised by high estradiol relative to progesterone—may be closer to a threshold at which weak membrane polarisation produces measurable facilitation. Conversely, an endocrine state associated with stronger GABAergic neurosteroid influence may attenuate, delay, or qualitatively alter stimulation effects. These examples remain mechanistic hypotheses, however, because cycle phase alone does not identify the causal hormone and because progesterone-related effects can depend on its metabolites, receptor sensitivity, and the direction of hormonal change. Importantly, hormones likely influence not only immediate excitability but also plasticity susceptibility—the capacity of the cortex to convert a brief perturbation into longer-lasting synaptic change. The Rivas-Grajales review highlights mechanistic pathways through which oestrogen may be plasticity-permissive: oestrogen increases excitability partly via NMDA-related mechanisms and reduces GABAergic responses, and oestrogen administration increases BDNF expression in the prefrontal cortex in animal models, a key molecular mediator of synaptic plasticity. These converging lines imply a principled bridge to tDCS: if anodal tDCS aftereffects depend substantially on NMDA-linked and BDNF-supported processes, then high-oestrogen and high-testosterone milieus should, in general, increase the probability and magnitude of excitatory aftereffects, whereas high-progesterone milieus should reduce them by strengthening inhibition and potentially raising the effective threshold for LTP-like change [71].

The review also supports a metaplasticity interpretation of apparent “sex effects” in stimulation studies: rather than representing a stable trait difference between men and women, sex-linked variability can reflect state-dependent changes that modulate whether stimulation produces detectable plastic responses at all. A particularly relevant example is the prefrontal TMS–EEG work summarised in the review, where only females in a high-oestrogen phase demonstrated significant changes in TMS-evoked potentials after prefrontal rTMS, and oestrogen was argued to influence not just mean effects but the magnitude and variance of stimulation-induced plasticity responses. Although that finding comes from rTMS rather than tDCS, the logic generalises to tDCS because both are forms of noninvasive neuromodulation whose lasting effects rely on the excitatory–inhibitory milieu and plasticity mechanisms of the cortex. Under this view, a mixed-sex tDCS analysis that collapses across hormonal context may occasionally detect sex × stimulation interactions not because “female sex” is the causal moderator, but because a subset of women happen to be in a high-oestrogen (or high E/P ratio) state that makes neuromodulation effects larger, more reliable, or differently expressed.

Menstrual-cycle physiology provides a biologically plausible “hidden moderator” that can inflate within-group variance in women and thereby destabilise apparent sex moderation across studies. The Rivas-Grajales synthesis shows that cycle-related changes can be robust for intracortical facilitation/inhibition metrics even when motor threshold itself is sometimes unchanged across cycle phases in healthy women. This distinction is important for tDCS interpretation: stability in a coarse excitability measure (e.g., MT) does not imply stability in the specific inhibitory and facilitatory microcircuit dynamics (e.g., SICI/ICF balance) that govern whether weak polarising currents produce meaningful behavioural or physiological aftereffects. In practice, if women in a given tDCS study are sampled across heterogeneous cycle phases without hormone measurement, the female group becomes a mixture of participants in more excitable, more plasticity-permissive states (late follicular/ovulatory) and participants in more inhibited states (mid-luteal). That mixture can wash out stimulation effects in women, produce inconsistent directionality across samples, or create seemingly idiosyncratic sex-by-stimulation interactions that are actually driven by unmeasured endocrine context [71].

A further nuance emphasised in the review is that the “progesterone is inhibitory” mapping is not universal; clinical populations can show altered neurosteroid physiology such that hormonal phase may be associated with different excitability signatures than in healthy controls. In epilepsy and PMS, higher cortical excitability has been observed during the high-progesterone luteal phase, a pattern plausibly linked to altered GABA_A receptor sensitivity or neurosteroid withdrawal/tolerance effects. This is highly relevant to tDCS because clinical tDCS cohorts often include individuals with perturbed inhibitory circuitry or altered neuromodulatory tone. In such contexts, sex-linked and hormone-linked excitability differences may not mirror those in healthy populations, and apparent sex moderation in tDCS outcomes could reflect higher-order interactions among sex, endocrine state, and disorder-specific shifts in inhibitory signalling rather than sex alone.

Finally, the Rivas-Grajales review makes the translational point that exogenous hormonal regimens—such as those used in in vitro fertilisation, hormone replacement therapy, and gender-affirming treatment—are frequently delivered at higher-than-physiologic doses and may therefore exert larger effects on cortical excitability than typical menstrual cycle variation. For tDCS, this implies that “gender effects” may be especially pronounced, or especially variable session-to-session, in participants undergoing rapid hormonal transitions or high-dose hormonal treatment, because the baseline excitability set-point and plasticity susceptibility may shift more dramatically than would be expected under standard assumptions [71]. Taken together, the most defensible mechanistic integration is that many apparent gender differences in tDCS outcomes can be understood as the product of hormone-sensitive differences in E:I balance and stimulation-induced plasticity susceptibility, with oestrogen and testosterone tending to bias toward greater excitability and facilitation, and progesterone tending to bias toward greater inhibition, while acknowledging that disorder states can invert or distort these mappings. This framework provides a concrete physiological basis for why sex/gender may appear as a moderator in tDCS studies even when the device settings are identical, and why controlling or measuring endocrine context is a critical step in interpreting and stabilising “gender effects” in neuromodulation results.

### 4.2. Menstrual Cycle and Clinical Contexts

Across the menstrual cycle, the key physiological driver for neuromodulation-relevant variability is not “female sex” per se but the shifting balance between estradiol-dominant and progesterone-dominant states, which measurably alters cortical excitability, inhibition, and plasticity. In healthy, naturally cycling women, converging TMS evidence shows that cortical excitability is state-dependent across cycle phases: Smith et al. provided early direct physiological evidence that menstrual phase changes cortical network excitability, with stronger inhibition in the luteal phase than the follicular phase using paired-pulse paradigms sensitive to GABAergic tone [72]. This general pattern aligns with the mechanistic summary in Rivas-Grajales et al., which synthesises multiple studies indicating that higher oestrogen is associated with greater excitability (e.g., greater ICF, larger MEPs), whereas higher progesterone is associated with greater inhibition (e.g., stronger SICI, longer silent periods) [71]. Smith et al. (2002) further framed this in neurobiological terms that map cleanly onto neuromodulation mechanisms: estradiol enhances excitatory neurotransmission, while progesterone-derived neurosteroids increase GABAergic inhibition, and the paired-pulse TMS response is sensitive to agents that alter these glutamatergic and GABAergic systems [73]. Inghilleri et al. then demonstrated cycle-linked differences in rTMS response profiles in women, showing that MEP size increased during stimulation at ovulation (day ~14; high oestrogen) but not during menses (day ~1), supporting the inference that low-oestrogen states reduce excitability and that oestrogen-linked changes can shape how the cortex responds to repeated stimulation trains [74]. The Rivas-Grajales review integrates similar findings and emphasises the recurring signature of higher facilitation around high-oestrogen phases and stronger inhibition around high-progesterone phases, even when gross thresholds are sometimes unchanged—a crucial nuance, because stability in motor threshold does not necessarily imply stability in intracortical inhibition/facilitation balance, which is often the more relevant substrate for plasticity expression [71]. Importantly, cycle phase can modulate not only baseline excitability but also the plastic response to stimulation: Rogers & Dhaher reported that female sex hormones modulate the response to low-frequency rTMS, reinforcing that endocrine state can alter induced aftereffects rather than merely shifting baseline measures [75]. Consistent with this “hormone-as-gain-control” framing, Rivas-Grajales et al. highlight prefrontal stimulation work where only women in a high-oestrogen phase showed robust stimulation-linked changes in TMS–EEG evoked potentials, and oestrogen appeared to influence both the magnitude and variability of stimulation-induced plastic effects. Mechanistically, this is coherent with the review that oestrogen increases excitability through enhanced glutamatergic transmission and reduced GABAergic responses, while progesterone exerts inhibitory actions via GABA_A modulation [71]; it is also consistent with evidence that ovarian steroid milieu can alter cortical inhibitory neurochemistry and related physiological readouts across the cycle, including MRS findings suggesting menstrual phase–linked differences in cortical GABA function (and abnormal phase-dependent patterns in PMDD) [76]. Taken together, these studies justify treating menstrual phase (and, ideally, measured hormone levels) as a biologically plausible “hidden moderator” that can increase within-women variance, change responsiveness to stimulation, and thereby create or obscure apparent sex/gender interactions when women are analysed as a single undifferentiated group.

Clinical contexts further strengthen the argument that endocrine state can meaningfully reshape excitability in ways directly relevant to interpreting variability in neuromodulation outcomes, because several disorders show altered or even inverted hormone–excitability mappings relative to healthy physiology. While progesterone is typically associated with increased inhibition in healthy participants (e.g., increased SICI and inhibitory signatures in the luteal phase), women with premenstrual syndrome show evidence of an abnormal brain response to luteal progesterone: in paired-pulse TMS, control subjects demonstrated the expected luteal increase in inhibition, whereas women with PMS exhibited relative facilitation during the luteal phase, interpreted as physiological evidence for atypical progesterone/neurosteroid modulation of cortical circuits [77]. Similarly, catamenial epilepsy provides a clinically salient example where luteal-phase physiology can be associated with cortical hyper-excitability rather than inhibition; TMS studies explicitly examining menstrual-cycle changes in women with catamenial epilepsy have reported altered excitability patterns across cycle phases, supporting the idea that disorder-linked changes in GABAergic signalling and neurosteroid sensitivity can reverse expected effects [78,79]. Rivas-Grajales et al. directly underscore this point, noting that the opposite pattern (higher excitability with higher progesterone in the luteal phase) has been observed in epilepsy and PMS, likely reflecting altered GABA neurotransmission and neurosteroid dynamics in these populations. This has direct interpretive consequences for neuromodulation studies that include clinical samples: if a disorder shifts the hormone–inhibition relationship, then cycle phase may not merely add “noise,” but can qualitatively change the direction of excitability and plasticity susceptibility during stimulation sessions. In mood disorders, endocrine context also matters because sex differences in disease prevalence and symptom fluctuations intersect with hormonally driven physiology [71]. Rivas-Grajales et al. emphasise that women have higher rates of depression than men and that discrete reproductive-hormone transition periods—premenstrual, postpartum, and menopause—are linked to elevated vulnerability, with the menopause transition described as a particularly vulnerable period for onset or exacerbation of depression. The review also highlights clinical evidence that ovarian steroid milieu relates to antidepressant response in stimulation contexts: in treatment-resistant depression treated with prefrontal rTMS, higher estradiol-to-progesterone ratios in premenopausal women were associated with greater clinical improvement, supporting the idea that an oestrogen-dominant (or higher E/P ratio) milieu may be more plasticity-permissive and more responsive to interventions that rely on synaptic potentiation mechanisms [71]. In severe brain injury, endocrine state has also been linked to stimulation-linked recovery signals; for instance, higher estradiol levels correlated with improvement in level of consciousness after a prefrontal rTMS course in men with severe TBI, illustrating that even outside female reproductive cycling, sex hormones can act as clinically meaningful physiological modifiers [71]. Finally, beyond endogenous cycling, exogenous hormonal contexts are likely to be particularly important for interpreting variability because they can shift hormones to supraphysiological levels: Rivas-Grajales et al. explicitly caution that iatrogenic hormonal treatments (e.g., IVF protocols, hormone replacement therapy, gender-affirming hormone regimens) involve higher-than-physiologic dosing and therefore may more dramatically affect neurophysiology than typical menstrual variation. In practical terms, that means that “gender effects” observed in neuromodulation outcomes can plausibly reflect a composite of (i) cycle-phase shifts in excitation/inhibition, (ii) disorder-specific alterations in neurosteroid responsiveness (especially in PMS/PMDD and catamenial epilepsy), and (iii) larger endocrine perturbations from treatment states such as menopause therapy or gender-affirming care, all of which have documented links to TMS-based excitability measures and, by extension, to the physiological substrate that determines how reliably stimulation-induced plasticity is expressed [71].

### 4.3. Why Modelled “Sex Differences in Dose” Do Not Map Cleanly onto Behavioural “Sex Differences”

Computational head models can show statistically reliable sex-linked shifts in delivered electric field (E-field) or current density under identical device settings, but several mechanistic “translation gaps” make it unsurprising that these dose differences do not map one-to-one onto behavioural outcomes.

#### 4.3.1. “Modelled Dose” Is a Physical Quantity; “Biological Dose” Is the Field Component That Actually Polarises Task-Relevant Neurons

Finite-element models typically report E-field magnitude or ROI-averaged summaries, yet the neural effect depends strongly on the component of the field relative to cortical geometry (e.g., normal vs. tangential to the cortical sheet) and on neuronal orientation. Work on determinants of E-fields in realistic head models emphasises that cortical folding, tissue conductivities, and orientation of cortical columns shape which field components dominate locally [80,81,82]. Consistent with this, evidence from motor physiology shows that tDCS effects can be specific to which corticospinal inputs/circuits are engaged (e.g., orientation/circuit specificity), implying that two people with similar mean E-field magnitudes could still receive meaningfully different effective polarisation of the circuits that matter for a given task [82,83]. So, even if one sex shows higher average E-field in an ROI, the behaviourally relevant field component for the recruited circuit may not differ in the same direction (or at all), especially when folding/orientation differs across individuals.

#### 4.3.2. Small Physical Dose Offsets Are Filtered Through Strong State Dependence and Circuit Recruitment

tDCS is widely understood as subthreshold neuromodulation: it biases membrane potentials and synaptic efficacy rather than directly driving activity, so behavioural impact depends on what networks are active and plastic at the time of stimulation [8,83,84]. If a task engages different strategies, subnetworks, or learning phases across people, then the same delivered E-field can yield different outcomes (including null effects) because the “lever” being modulated is not the same. This helps explain the current review’s repeated observation that sex moderation often emerges in late phases, high-demand conditions, or trajectory measures—times when the engaged circuitry and plasticity regime are most constrained.

#### 4.3.3. Dose–Response Is Not Reliably Linear in Humans at Conventional Intensities, So Anatomical Dose Differences Need Not Produce Proportional Behavioural Differences

Multiple physiological studies indicate that excitability after-effects do not scale monotonically with intensity, and that polarity–intensity relationships can even reverse under some parameter regimes (e.g., cathodal at higher intensities showing facilitation rather than inhibition) [85,86,87]. Reviews and empirical work therefore caution against assuming that “more E-field” straightforwardly implies “more behavioural effect” [83,88]. If one sex is, on average, shifted slightly along a nonlinear curve (or distributed differently around a threshold), the resulting behavioural differences can be inconsistent across tasks, targets, and intensities—even when modelled E-field differences replicate.

#### 4.3.4. Inter-Individual Variability Is Often Larger than Sex-Linked Mean Differences, and Behavioural Endpoints Add Additional Noise

A consistent finding in physiology is large between-person variability in response to standard tDCS protocols, with substantial fractions of “non-responders” depending on outcome definition [89,90]. Meanwhile, modelling studies and dose-control papers show wide spreads in estimated E-fields across individuals under the same montage, motivating individualised dosing approaches [91,92,93]. In this setting, a modest sex-linked shift in mean modelled dose can be overwhelmed by within-sex variance, and behavioural measures (accuracy, RT, symptom scales) typically have lower signal-to-noise than physiological readouts, further weakening dose → behaviour mapping.

#### 4.3.5. Modelled E-Fields Can Predict Outcomes in Some Contexts—But Typically When the Model Targets the Right Field Metric and the Outcome Is Mechanistically Proximal

There is growing evidence that individualised E-field estimates (often specifically the field component normal to the cortical surface) can explain meaningful portions of variability in physiological or connectivity outcomes and, in some cases, behavioural response [94,95,96]. At the same time, such predictive relationships are not universal, and reviews highlight limits to assuming linear, directly behavioural dose–response relations at typical field strengths [83,88]. A sex difference in generic dose summaries (e.g., mean E-field in a broad ROI) may fail to translate unless it also produces a sex difference in the mechanistically relevant field metric for the engaged circuit and outcome.

### 4.4. Anatomy as a Dose-Shaping Mechanism: Scalp-to-Cortex Distance, Skull Layers, and Cortical Geometry

Anatomy may contribute to apparent sex effects by altering the cortical electric field produced by a fixed current. These anatomical influences are spatially specific and depend on scalp location, montage, and target. They should therefore not be interpreted as producing a uniform global effect. Three anatomical families are especially dose-relevant.

#### 4.4.1. Scalp-to-Cortex Distance Is Not a Single Number; It Is a Location-Specific Stack of Tissues That Jointly Filters Current

What is often casually described as “scalp-to-cortex distance” (SCD) comprises multiple compartments—skin/soft tissue, compact skull bone, spongy (diploë) bone, CSF, and then cortex. Each layer differs in conductivity, thickness, and spatial heterogeneity, so two heads with the same total SCD can still produce different intracranial fields depending on which layer “accounts” for that distance. Modern modelling work shows that these anatomical features systematically reshape E-field magnitude and distribution, and that the resulting E-field can vary substantially even under identical device settings [81,96,97].

Recent large-scale tissue-thickness mapping across many channels reinforces that the components of SCD vary meaningfully with age and sex across the scalp, and that differences are not uniform across locations—supporting the empirical pattern in current results that sex moderation depends strongly on stimulation site and montage [98].

#### 4.4.2. Skull Layers Matter Because They Are a Major Resistive Barrier—And Their Thickness and Composition Vary

Finite-element analyses that explicitly separate skull layers (compact vs. spongy/diploë) demonstrate that skull microstructure is not a trivial detail: it changes current shunting and can re-weight where the field concentrates in cortex [81,99].

Independent cranial-anatomy studies report that cranial vault thickness and its age trajectory can differ by sex, and that diploë thickness shows region-specific patterns (including frontal-region differences in some datasets) [100,101]. Mechanistically, this matters because thicker or differently proportioned skull layers can reduce field strength at cortex and increase superficial voltage drop, potentially shifting the balance between cortical engagement and peripheral (skin/nerve) co-stimulation—an interpretation that aligns with current results emphasising montage- and intensity-dependent sex differences in sensation and efficacy.

#### 4.4.3. CSF Thickness Is a Dominant “Field Shaper” Because It Is Highly Conductive and Spatially Irregular

Among anatomical predictors, CSF thickness and distribution repeatedly emerge as major drivers of inter-individual variability in modelled E-fields. In motor-cortex modelling, CSF thickness was reported as a primary factor explaining a large share of between-person variance in the induced field, because a thicker CSF layer can effectively redistribute current and reduce focal field strength at the cortical surface [97]. This is a key bridge to Section Individual Variability and Weak Cross-Site Reliability in Delivered Dose: even if sex-linked mean differences exist in some tissue compartments, within-sex CSF variability can be large enough to swamp mean shifts, helping explain why modelled sex effects do not consistently translate into behavioural sex effects unless the task, montage, and endpoint are positioned near a functional threshold.

#### 4.4.4. Cortical Geometry Determines Whether the “Relevant” E-Field Reaches the Circuits That Control the Task

Finally, even if two individuals have similar average ROI E-field magnitudes, differences in gyral/sulcal anatomy can change the orientation and local concentration of the field at the cortical surface. Determinant analyses emphasise that folding patterns and tissue boundaries shape not only how much field arrives, but also the field’s spatial gradients and direction relative to cortical columns—features that are plausibly more biologically relevant than a bulk ROI average [81,96,102]. This provides a mechanistic rationale for the interaction-heavy patterns (sex × montage × target × endpoint): even small anatomy-linked shifts can redirect the field toward different subregions or orientations, producing qualitatively different effects depending on which network component a task actually recruits.

Sex should not be interpreted as directly causing a uniform difference in response. Instead, sex-linked and age-linked anatomy may alter the distribution of the delivered electric field in montage- and site-specific ways. Skull-layer composition and CSF geometry are likely contributors. These anatomical differences may make sex moderation more likely for some targets and montages than for others.

### 4.5. Individualised Dosing as a Test of Mechanism (Not Just a Methodological Tweak)

A key interpretive advance in the results is that individualised dosing can be used to interrogate why apparent sex moderation occurs, not merely to “improve” protocols. Conceptually, individualised dosing turns a broad, correlational label (sex) into a testable causal chain: anatomy → delivered cortical E-field at the target → physiological change → behavioural/symptom change. If sex differences in outcomes are partly driven by systematic differences in delivered dose under fixed mA, then scaling current to equalise a mechanistically relevant E-field metric at the target should attenuate sex associations—exactly the pattern implied by current dose-equalisation findings.

#### 4.5.1. Why Dose Control Is a Mechanistic Experiment

Fixed-dose tDCS (e.g., “2 mA for 20 min”) implicitly assumes that the same nominal current yields comparable intracranial stimulation across participants. However, individual head anatomy produces large variation in predicted E-field strength at the cortical target, even when montage and device settings are identical. This is one of the strongest, most replicated modelling claims in the broader tDCS literature and is now widely recognised as a primary source of heterogeneity [93,103,104].

“Dose-controlled” or “E-field–controlled” tDCS reframes the intervention from current at the electrodes to field at the cortex. In practice, it estimates each participant’s required scalp current to reach a pre-specified target-field strength (or target metric) at the intended cortical ROI. A foundational demonstration showed that applying a single fixed current produces wide variability in E-field at the target, whereas individualising current based on models can reduce that variability at the cortical site [93].

Mechanistically, this is not merely a variance-reduction strategy. It is a mediation probe: if a putative moderator (including sex) operates mainly through delivered E-field differences, then removing E-field differences should remove—or sharply reduce—the moderator’s explanatory power. If, instead, sex moderation persists after E-field equalisation, that points away from a simple “dose partitioning” account and toward alternative mechanisms (endocrine state, baseline excitability, strategy/learning, peripheral nerve co-stimulation, etc.).

#### 4.5.2. What Exactly Is Being “Equalised,” and Why the Choice Matters

A common pitfall is to treat “E-field at target” as a single quantity. In reality, different modelling pipelines and papers operationalise target engagement differently (peak vs. mean, ROI-averaged vs. voxelwise, magnitude vs. directional components). Determinants work shows that orientation relative to cortical geometry (e.g., normal-to-surface components) can change which circuits are effectively polarised, so equalising a coarse ROI mean may not equalise the biologically relevant field component for the task [104,105].

This matters for interpreting sex moderation specifically: sex-linked anatomy might shift where within an ROI the field concentrates or the dominant orientation of the field, so two participants could have matched mean E-field but still differ in circuit-level engagement. Dose-control designs therefore function best as mechanistic tests when they (i) specify a biologically motivated E-field metric, and (ii) verify downstream engagement with physiological or neuroimaging readouts when feasible.

#### 4.5.3. Individualisation Without MRI: “Physiology-Guided” Dosing as an Alternative—And a Falsification Tool

A practical barrier to model-based dosing is structural MRI. Several groups have therefore pursued MRI-free or hybrid approaches that use physiological thresholds as proxies for individual current-flow differences. One influential method uses reverse-calculated E-field modelling to estimate how much scalp current would be needed to achieve a desired cortical field (e.g., 1 V/m) and then explores whether inexpensive physiological measures can predict that individualised dose. In a representative study, transcranial electrical stimulation motor threshold (TES MT) showed promise as an MRI-free correlate of reverse-calculated dose, underscoring that individualised dosing can potentially be implemented without full imaging in some settings [92].

At the same time, physiology-guided “individualisation” is itself a mechanistic test: if an excitability proxy (e.g., motor threshold–derived scaling) fails to improve consistency, that suggests that the proxy is not capturing the relevant source of variability for that montage/target/outcome, or that non-dose factors dominate. For example, attempts to individualise tDCS intensity based on baseline corticospinal excitability measures have not consistently improved responsiveness, highlighting that not all plausible personalisation rules actually target the key causal bottleneck [106].

#### 4.5.4. Dose Standardisation at Scale: From “Personalisation” to “Transportable Correction”

Recent work is also moving beyond one-off individualised pipelines toward population-level dose standardisation, where demographic/morphological predictors are used to estimate expected E-field and adjust dosing across large samples and multiple montages. A 2025 study trained regression models to predict peak E-field strengths across multiple montages in hundreds of adults and then used those predictions to standardise transcranial electrical stimulation dosage [107]. This direction matters for the sex question: it offers a pragmatic route to reducing systematic group differences in delivered dose (including sex-linked anatomical shifts) without requiring MRI for every participant—while still making “dose equalisation” explicit and testable.

#### 4.5.5. Limits: What Individualised Dosing Can—And Cannot—Explain About Sex Moderation

Even perfectly executed E-field dosing would not eliminate sex moderation if the primary driver is state-dependent neurobiology (e.g., estradiol/progesterone effects on inhibition/excitation balance), task strategy, or peripheral co-stimulation that differs across individuals. Dose control is therefore best viewed as a mechanism-separating intervention: it can isolate how much of a sex association is attributable to delivered cortical field differences versus downstream physiological or behavioural factors.

In this sense, individualised dosing is not a cosmetic upgrade. It is a way to convert a heterogeneous literature into sharper inferences: when sex effects shrink after E-field equalisation, the evidence supports a dose-mediated account; when they persist, the field is forced to look to non-dose mechanisms.

### 4.6. Why Higher Intensities Can Amplify Sex-Linked Differences: Nonlinear Neurophysiology and Peripheral Co-Stimulation

Results show that sex moderation is often most detectable at the upper end of intensity (notably ~4 mA), where subjective sensation differences become larger and behavioural separation is easier to observe. Two mechanism families make this “high-intensity amplification” pattern expected: (i) nonlinear, state-dependent neurophysiology and (ii) increasing peripheral (cutaneous/nerve) co-stimulation that can differentially bias experience, blinding, and strategy.

#### 4.6.1. Nonlinear Cortical Physiology: Intensity Can Change the Kind of Plasticity, Not Just Its Size

A common interpretive error is to assume that raising intensity simply scales the same biological effect. In human motor physiology, however, tDCS after-effects show nonlinear dose–response relationships and can even reverse with intensity. A canonical example is the finding that cathodal tDCS at 2 mA (20 min) produced facilitation-like after-effects rather than the inhibitory pattern typically associated with lower-intensity cathodal stimulation, demonstrating that polarity rules are not invariant across intensities [108]. Systematic intensity “titration” studies confirm that after-effects across multiple intensities (e.g., 0.5–2.0 mA) are often non-monotonic for both anodal and cathodal stimulation, consistent with the idea that the induced plasticity regime shifts as dose changes [87]. Work extending cathodal dosing into the 1–3 mA range similarly reports dose- and duration-dependent nonlinearities, reinforcing that higher intensity can change response direction or timing rather than simply “more inhibition” [85]. Pharmacological mechanistic work links these nonlinearities to calcium-dependent plasticity dynamics, supporting the plausibility that small differences in baseline excitability (or delivered field) can push individuals into different net plasticity outcomes at higher intensities [109].

Why does this matter for sex-linked differences? If the underlying dose–response curve has bends, thresholds, or sign reversals, then even modest sex-linked shifts in delivered E-field (via anatomy) or baseline excitability (via hormones/state) can move men and women into different parts of that curve. Under such conditions, group differences can become more visible at higher intensity because that is where nonlinearities are strongest and where “which regime you’re in” matters most.

#### 4.6.2. Peripheral Co-Stimulation Grows with Intensity and Can Bias Behaviour Through Sensation, Attention, Expectancy, and Blinding

As current increases, so does electric field strength in the skin and the probability of exciting cutaneous afferents. Work on the electrode–skin interface emphasises that perceived tingling, itching, or burning during tDCS is most parsimoniously explained by peripheral nerve activation and electrochemical processes, and that “hot spots” of current density (e.g., near electrode edges or skin inhomogeneities) can increase discomfort even when average current density is controlled [18,110].

Two empirical points are especially relevant. First, electrode montage/geometry alters cutaneous sensation at a given nominal dose. For example, discomfort depends on electrode size and interface properties; large electrodes can produce greater discomfort than smaller ones even at equivalent current density, showing that peripheral experience is not a trivial linear function of “mA” [110,111]. Second, peripheral pathways can generate central effects. In transcranial alternating current stimulation (tACS), careful experiments demonstrate that some motor-system effects can be mediated by transcutaneous stimulation of peripheral nerves rather than direct cortical entrainment, illustrating a plausible “peripheral-to-central” route for behavioural modulation when skin fields are high [112,113].

Although this peripheral-mechanism evidence is strongest for tACS, the core implication generalises to high-intensity tDCS: as intensity rises, peripheral afferent input becomes more salient and more capable of influencing arousal, attention, and decision strategy—even if the intended cortical modulation is similar.

Why does this matter for sex-linked differences? Peripheral co-stimulation creates several plausible amplification channels that do not require sex differences in cortical response per se. If one sex systematically experiences stronger sensation (for anatomical or psychophysical reasons), then blinding integrity, distraction, and expectancy can diverge more at higher intensities, increasing the chance of sex × stimulation interactions in demanding tasks. Independent pain research shows that, on average, women exhibit greater pain sensitivity and higher prevalence of clinical pain, and sex differences in pain inhibition are documented across experimental and epidemiological literatures [114,115]. Placebo/nocebo literatures also suggest sex-linked differences in expectation-driven symptom reporting in some contexts, with reviews reporting patterns of stronger nocebo responding in females in subsets of studies [116].

Taken together, these bodies of evidence make a specific, testable prediction that fits current results: sex-linked differences should be smallest at conventional intensities where sensation is mild and blinding is robust, but should grow as intensity increases because both nonlinear cortical dynamics and peripheral-afferent pathways become more influential.

#### 4.6.3. Practical Implication for Interpreting High-Intensity Sex Moderation

At higher intensities, sex moderation is more plausibly multi-causal: nonlinear plasticity regimes increase the chance of true neurophysiological divergence, while peripheral co-stimulation increases the chance of experience-/expectancy-mediated divergence. The strongest inference strategy, therefore, is not to treat high-intensity sex effects as “neural” or “artefact,” but to design studies that can separate the two—e.g., richer sensation time-course measures, blinding confidence, peripheral nerve controls (topical anaesthesia or optimised interfaces), and mechanistically proximal biomarkers alongside behaviour.

### 4.7. Sensation, Expectancy, and Blinding: Plausible Mediators at High Intensity, Insufficient as a Universal Explanation

A credible interpretation of the high-intensity findings in Section 3 is that sex-linked differences in subjective experience (tingling, burning, itching, discomfort) can sometimes mediate sex-by-stimulation effects—especially when stimulation is strong enough that sensations are salient and sham mimicry becomes difficult. This mediation pathway is well supported by the broader sham-validation literature: at 2 mA, participants and assessors can often discriminate active from sham above chance, and sensory cues such as itching and skin redness are implicated as discriminators [117,118,119,120]. Importantly, the field has also recognised that “sham” is not a single standardised entity: reviews argue that heterogeneity in sham protocols can introduce both blinding variability and, potentially, small biological effects that further complicate inference [121,122].

Why can these experiential factors produce sex-moderated behavioural outcomes at higher intensities?

Once sensations become noticeable, they can influence outcomes through at least three mechanisms that do not require any true sex difference in cortical responsiveness.

First is unblinding and expectancy. If participants infer condition, expectancy can shift effort, caution, and strategy—effects that are especially impactful in high-demand tasks. Evidence from placebo/nocebo research indicates sex-linked differences can occur in expectancy-driven responding (with patterns that vary by paradigm and induction method), making it plausible that unblinding could translate into sex-contingent behavioural changes in some designs [116,123].

Second is attentional capture/distraction. Salient sensations can draw attention away from the task or increase arousal, disproportionately affecting performance when cognitive control is taxed.

Third are peripheral-to-central pathways. Beyond “nuisance,” peripheral stimulation can contribute to central effects; contemporary discussions of transcranial electrical stimulation increasingly emphasise that cranial/cervical nerve co-stimulation may explain part of what is attributed to direct cortical modulation, motivating designs that explicitly suppress peripheral input (e.g., topical anaesthetics) when feasible [124,125,126,127].

These mediators become more plausible at higher intensities because sensory differences are larger and sham strategies are more strained. Even at conventional intensities, there is evidence that blinding can be compromised (depending on population, sham method, and assessment), so at ~4 mA the risk is naturally amplified unless sham is engineered to match the full sensory time course [120,121,122].

#### 4.7.1. Why “It’s Just Unblinding/Sensation” Is Not a Sufficient General Explanation?

At the same time, several considerations argue against treating sex moderation as merely an artefact of discomfort or blinding failure across the literature.

Blinding problems are not synonymous with expectancy effects. Above-chance guessing does not guarantee that expectancy meaningfully drove performance; many studies do not measure expectancy strength, belief confidence, or moment-to-moment sensation trajectories, so a “blinding failed → outcome is demand characteristics” inference is often underdetermined. Reviews explicitly recommend richer blinding assessments because end-of-study guesses can miss when and how unblinding occurs [121,128].

Improved sham methods can preserve blinding even at higher currents, yet behavioural/physiological effects can still occur. Work developing and validating sham approaches for HD-tDCS (including higher amplitudes) reports that tailored sham waveforms can maintain blinding in some contexts, implying that high current does not inevitably break blinding [129,130].

Improved sham methods can preserve blinding even at higher currents, yet behavioural/physiological effects can still occur. Work developing and validating sham approaches for HD-tDCS (including higher amplitudes) reports that tailored sham waveforms can maintain blinding in some contexts, implying that high current does not inevitably break blinding [125,127].

#### 4.7.2. Sex-Linked Psychophysics Strengthens the “Plausible Mediator” Claim but Does Not Make It Universal

We noted earlier that independent pain research consistently reports that women, on average, show greater pain sensitivity and higher prevalence of many clinical pain conditions, and reviews also describe sex differences in pain modulation [114,131]. These differences make it plausible that, when stimulation becomes intense enough to produce stronger cutaneous sensations, women and men could diverge in distraction, discomfort-related strategy shifts, or nocebo-like responses. Yet placebo/nocebo reviews also emphasise that sex effects are context-dependent and not uniform across domains or induction methods, which again mirrors current thinking that sex moderation is interaction-structured rather than global [116,123].

#### 4.7.3. Net Interpretation for This Review

Sensation, expectancy, and blinding are most defensible as partial mediators of sex-linked differences under conditions where stimulation is more perceptible (higher intensity, longer ramps, skin redness) and where task demands make performance especially vulnerable to attentional capture or belief effects. However, because (i) sham variability is itself a complex source of heterogeneity, (ii) improved sham can preserve blinding even at higher currents, and (iii) peripheral and cortical mechanisms can plausibly operate in parallel, these experiential factors cannot serve as a universal explanation for sex moderation. The most rigorous path forward is therefore not to dismiss sex effects as artefacts, but to measure and model the relevant mediators (sensation time course, expectancy strength, blinding confidence) and, when feasible, incorporate peripheral-suppression controls (e.g., anaesthetic interfaces) or sham designs that better match the sensory profile across the full session.

### 4.8. Why Sex Moderation Concentrates in Demanding Tasks and Late-Phase Dynamics

Sex moderation was rarely a simple baseline offset. It tended to emerge when tasks became maximally demanding (high load, high conflict, uncertainty) and/or when outcomes were quantified over trajectories (training gains, late-block performance, delayed after-effects). This pattern is expected from the core properties of tDCS. It is a small, state-dependent neuromodulation with plasticity that unfolds over minutes to days, interacting with ceiling/headroom and strategy shifts that become most visible under stress.

#### 4.8.1. State Dependence Makes “Difficulty” a Gain Control for tDCS Effects

tDCS effects are interactive. The same stimulation can produce different (even opposite) effects depending on the engaged brain state, baseline performance, and task context. A review focused on individual differences explicitly highlights task difficulty as a driver of state-dependent responsiveness (i.e., difficult tasks), where stimulation effects can become detectable. This is because the system is already strongly recruited and closer to performance limits [132].

This is consistent with neuroimaging evidence showing that tDCS can modulate brain activity in a polarity- and brain-state–dependent manner, with different signatures at rest versus during task engagement [133]. In other words, high-demand tasks are not merely “harder”; they shift the cortex into a different dynamical regime (higher control recruitment, stronger top-down coupling), creating more opportunity for a weak perturbation to bias network competition.

If men and women differ in baseline strategy, arousal, or endocrine state (even subtly), demanding contexts are precisely where those baseline differences matter most—because the system is operating near constraints where small shifts in control allocation or noise suppression can change which strategy “wins”.

#### 4.8.2. Headroom and Ceiling Effects: Stimulation Needs Room to Move Behaviour

tDCS effects on behaviour are typically small-to-moderate, especially in healthy samples, so ceiling performance can mask genuine physiological modulation. Difficult tasks create headroom (avoid ceiling accuracy; expand RT variability), increasing sensitivity to any stimulation-induced shift in efficiency or control. This is one reason why many effects in the Results appear specifically at maximal loads or conflict conditions rather than at low demand.

A complementary point is that “baseline ability” can gate detectability: when performance is already high, behavioural readouts may not capture underlying excitability changes; when performance is strained, the same excitability shift can translate into measurable differences in accuracy/RT and, importantly, in how performance changes across trials.

#### 4.8.3. Learning and Adaptation: Sex Moderation Becomes Visible in Trajectories Because Plasticity Is Cumulative

Many of the current effects appear in training gains, late blocks, or post-stimulation probes. That aligns with evidence that stimulation can preferentially influence learning dynamics and consolidation, not just momentary online performance.

A seminal multi-day study showed that noninvasive stimulation enhanced motor skill acquisition over multiple days through an effect consistent with consolidation mechanisms [134]. Even in shorter paradigms, learning stage matters: a study explicitly examining the interaction of task-related learning and tDCS effects on executive functions concluded that stimulation effects depended on participants’ experience/learning level in the task [135].

Why does this concentrate sex moderation in late phases?

Late-phase performance often reflects strategy stabilisation, reduced exploration, and greater reliance on the targeted control network (e.g., maintaining rules under fatigue, suppressing prepotent responses, optimising speed–accuracy trade-offs). If sex-linked factors (delivered dose differences, hormone-modulated excitability, or strategy preferences) change the rate or direction of adaptation, the divergence may only appear after sufficient trials for learning to differentiate groups.

#### 4.8.4. Delayed Neurochemical and Synaptic Mechanisms Fit Late and Post Effects

tDCS after-effects have been tied to synaptic plasticity mechanisms: classic physiology demonstrates that weak direct current stimulation can induce excitability changes that outlast stimulation [5]. Pharmacological work indicates that longer-lasting after-effects depend on NMDA receptor mechanisms [84].

At the neurochemical level, MRS studies show polarity-sensitive modulation of cortical neurotransmitters, including GABA changes with anodal stimulation [12]. Importantly for the “late-phase” theme, the magnitude of GABA decrease during anodal tDCS has been linked to motor learning and learning-related activity changes [136]. Together, these findings make it plausible that behavioural consequences, especially those tied to learning, inhibition, or strategy, emerge most clearly when the relevant plasticity processes have had time to accumulate (late blocks) or when after-effects are expressed (delayed probes).

Link to sex moderation is that any sex-linked factor that shifts excitation–inhibition balance (including endocrine state) can plausibly alter how strongly these plasticity-related processes are engaged, making sex moderation more visible in outcomes that index learning/consolidation rather than immediate online performance.

#### 4.8.5. Why Demanding Tasks Are “Where Differences Show Up” Rather than “Where Effects Are Bigger”

The key interpretation is not simply that hard tasks yield larger effects, but that they change what the outcome measures. Under low demand, performance may be dominated by perceptual speed or simple response execution; under high demand, performance increasingly reflects control allocation, conflict resolution, and adaptive policy updating. These are precisely the computations most likely to be biased by small changes in prefrontal/parietal network excitability. A weak perturbation can therefore appear as a sex × stimulation interaction only when the task engages the control process that differs (in baseline or susceptibility) between groups.

Sex moderation concentrates in demanding tasks and late phases because tDCS is state-dependent and its behaviourally relevant consequences often ride on plasticity and adaptation. High demand increases headroom and recruits the targeted control networks more strongly, while late-phase measures capture cumulative learning, consolidation, and delayed neurochemical after-effects. These are the conditions under which modest sex-linked differences, whether in delivered dose, excitability state, or strategy, are most likely to become detectable as interaction-structured effects rather than as baseline group differences.

### 4.9. Broader Context: Heterogeneity, Reproducibility, and Why “Sex” Should Be Modelled as a Proxy System

The sex-moderation patterns summarised in the results section sit inside a larger, field-wide reality: tDCS effects are heterogeneous, and that heterogeneity has tangible consequences for reproducibility and interpretation. Importantly, this does not mean tDCS “does nothing.” It means that many effects are small, state- and context-dependent, and highly sensitive to dosing geometry and participant biology, so subgroup findings (including sex moderation) can be both real and unstable unless studies explicitly model the underlying sources of variance.

#### 4.9.1. Heterogeneity Is a First-Order Feature of tDCS, Not a Nuisance

Across the broader literature, inter-individual variability in response is repeatedly emphasised as a defining characteristic of noninvasive brain stimulation. Large-scale and review work argues that responses can be multimodally distributed (e.g., “responders” and “non-responders”), and that reliability across sessions can be limited, particularly when outcomes are distal behavioural measures rather than proximal physiology [20,137,138].

In parallel, methodological guides and modelling reviews emphasise that seemingly “identical” tDCS protocols can produce meaningfully different intracranial fields because of anatomy and montage implementation details (electrode placement, contact quality, reference location), reinforcing that protocol labels (e.g., “2 mA anodal DLPFC”) are an imperfect proxy for delivered cortical engagement [18,137,139].

This broader heterogeneity is the backdrop against which sex moderation should be interpreted: sex can correlate with some biological determinants of current flow or excitability, but those determinants are only part of a large variance system.

#### 4.9.2. Reproducibility Concerns in Cognitive tDCS Make Subgroup Claims Easy to Over-Interpret

Meta-scientific evaluations have raised explicit concerns about the evidential strength of parts of the cognitive tDCS literature, especially for single-session effects in healthy adults. A quantitative review reported no reliable cognitive benefits from single-session tDCS across the evaluated domains [15].

A complementary “evidential value” analysis using p-curve methods concluded that samples of cognitive/working-memory tDCS studies showed minimal to no evidential value and estimated very low typical power in that literature subset—conditions that inflate the probability of chance significant findings and unstable interaction effects [140].

More broadly, reproducibility-focused commentary emphasises that typical sample sizes in tDCS studies are often too small for the modest effect sizes expected, and that underpowered designs are especially vulnerable when testing higher-order moderators (like sex × montage × condition interactions) [141].

The implication for current review is not that sex moderation findings are “artefacts,” but that the field conditions that already challenge main-effect reproducibility also challenge moderator reproducibility—so mechanistic interpretation should privilege convergent patterns (dose control, endocrine manipulation, trajectory effects) over isolated subgroup contrasts.

#### 4.9.3. The Field Response: Move from “Fixed mA” Protocols Toward Dose-Aware, Precision Frameworks

One concrete response to heterogeneity has been the push toward dose-aware stimulation, where the aim is to control (or at least quantify) the induced field at cortex rather than assume that nominal mA is comparable across participants. Reviews and technical guidance increasingly treat individualised modelling as a way to reduce unexplained variance and improve causal inference about brain–behaviour links [18,20,139]. This dose-aware shift matters for sex effects: it provides a principled way to test whether a sex association is primarily a delivered-dose artefact (anatomy → field differences → outcome) versus a true physiological susceptibility difference (e.g., endocrine modulation, E/I balance, learning strategy), because field equalisation selectively targets one path in that causal chain.

#### 4.9.4. Why “Sex” Is Best Treated as a Proxy System (And How to Model It Accordingly)

Our results repeatedly show that “sex effects” are rarely global; they are contingent on montage, target, intensity, outcome metric, and time course. In the broader context, that pattern strongly suggests that sex is functioning as an umbrella proxy for multiple partially correlated mechanisms, rather than as a unitary causal variable.

Several practical proxy categories are consistent with both the results and the wider literature.

Dose proxy: Sex correlates (imperfectly) with anatomical features that shape current flow; this contributes to sex-linked differences in delivered field under specific montages, but is easily overwhelmed by within-sex anatomical variability.

Plasticity-state proxy: Sex also correlates with endocrine milieus that can shift excitability/plasticity regimes across time within individuals, which can produce “sex differences” that are actually state-dependent. (This is exactly why measuring cycle status, contraceptive use, or hormone levels changes interpretability.)

Experience/expectancy proxy: Sex can correlate with pain sensitivity, side-effect reporting styles, and expectancy/nocebo susceptibility in some contexts, which becomes more behaviourally consequential when stimulation is perceptible, and tasks are demanding—yet this pathway will vary across populations and sham methods.

Cognitive/strategy proxy: Sex can correlate with baseline strategies or domain-specific experience distributions, which changes headroom and what performance metrics “mean” (e.g., exploration vs. exploitation, speed–accuracy policies), especially in late-phase learning.

Under this proxy view, the correct modelling stance is: do not ask whether sex “matters” in general; ask which components of the sex-proxy system are active in this protocol. Analytically, that argues for (i) including mechanistic covariates (modelled E-field metrics, baseline performance, hormone indices, sensation/expectancy measures) and (ii) treating sex not as a final explanation but as a flag that the study may be mixing multiple pathways.

## 5. Limitations and Future Directions

### 5.1. Participant and Sample Diversity

Existing tDCS studies of sex/gender effects have relied mostly on small, homogeneous cohorts. For example, modelling work used only 23–24 adults (∼12–13 of each sex) with narrow age ranges, and behavioural studies often recruit young, right-handed, healthy volunteers. Crucially, many samples lacked ethnic or age diversity—one study noted its subjects “appeared to be of European descent” and cautioned this likely does not capture the full range of cranial diversity. Moreover, even within mixed-sex samples there may be baseline performance or demographic differences. For instance, women in a reaching task were overall more accurate than men, and cognitive studies report faster or larger learning gains for one sex. Such baseline differences (or unmeasured factors like motivation) can confound the attribution of any tDCS-induced change to sex per se. Finally, sex differences may interact with age—one large modelling study found that young men had higher modelled current at a parietal site, whereas in older adults the pattern reversed (older women > older men). In short, narrow sampling limits generalisability and statistical power.

Future work should recruit larger, well-powered cohorts with balanced representation of sexes across a wide age range, as well as diverse ethnicities, handedness, and clinical backgrounds. Baseline measurements (e.g., motor speed, cognitive scores) should be collected and covaried, or used to stratify samples, so that stimulation effects are not conflated with pre-existing sex differences. Meta-analytic approaches or multisite consortia could help achieve adequate sample sizes to test sex-by-tDCS interactions robustly.

### 5.2. Modelling and Methodological Assumptions

Computational models of tDCS are indispensable for assessing sex-related differences in current flow, but they involve simplifying assumptions. Many finite-element head models assume isotropic conductivities and adopt literature-derived tissue values. In reality, tissue conductivity (especially skull bone) varies with composition and orientation: e.g., models that incorporated anisotropic skull conductivity found that it significantly alters the distribution of current in the brain (reducing the target vs. non-target ratio by ~12–14%). Few studies have integrated this complexity. Even high-resolution MRI models may not capture all relevant details—most segment only skin, skull, CSF, grey matter and white matter, often treating the skull as a single layer. Yet one study found that males and females differed in skull composition (dense cortical vs. cancellous bone) and this explained sex differences in modelled brain current. Models also typically omit hair, electrode-skin contact variability, and dynamic factors like blood flow or neurovascular coupling. As a result, predictions of current density are highly variable. For example, under identical settings, one model predicted a 10-fold spread across individuals, and within-subject differences across two scalp sites were essentially uncorrelated. These uncertainties limit confidence in any single model’s estimate of dose.

Modelling studies should progressively refine biophysical realism. This includes using subject-specific conductivities (e.g., via in vivo measurements), multi-layer skull models (cortical/sponge layers), and anisotropic white-matter tensors where possible. Electrodes should be modelled with realistic shapes and gels. Critically, models must be validated: combining tDCS with concurrent measurements (scalp potentials, EEG, or intracranial recordings) would help verify predicted currents. Sensitivity analyses should be reported to show how assumptions (conductivity values, segmentation differences) affect results.

### 5.3. Electrode Montage and Stimulation Parameters

Variability in montage (electrode placement, size, reference location) is another limitation. Different studies use sponge electrodes of various sizes, high-definition rings, or non-cephalic references without standardisation. Montage choice can interact with sex: for example, a ring (“HD”) montage at C3 produced a near threefold male advantage in modelled current, whereas other configurations attenuated that difference. Moreover, some montages (e.g., bilateral or ring configurations) may inadvertently stimulate cranial nerves or skin more than conventional bipolar setups. Indeed, authors have warned that “effects observed with ring or bi-cranial montages may partly reflect cranial nerve/scalp stimulation” rather than true cortical modulation. This is especially relevant for sex comparisons because females may experience more scalp sensation if less current reaches the brain, potentially biasing outcomes (e.g., by placebo or discomfort).

Future experiments should systematically compare montages and document their effects by sex. Reporting both scalp current density and modelled brain fields for each montage would clarify dose. Sham-controlled and double-blind designs are essential to control peripheral sensations. It may also be fruitful to personalise electrode montages (or current amplitudes) to match target brain dose across sexes or head sizes, then test behavioural effects under equalised dosing.

### 5.4. Hormonal and Physiological Factors

Hormonal-contraceptive use should not be treated as a single homogeneous category. Combined oral contraceptives differ in ethinyl estradiol or estradiol dose, progestin type, androgenic or anti-androgenic activity, monophasic versus multiphasic dosing, and active-pill versus withdrawal intervals. Progestin-only preparations create a different endocrine environment from combined preparations, and non-oral delivery systems may produce different pharmacokinetic profiles. Moreover, hormonal contraceptives suppress endogenous ovarian activity to different degrees, meaning that cycle-day labels have a different physiological interpretation in users and naturally cycling women. Therefore, a binary variable such as “contraceptive user versus non-user” cannot support a single mechanistic interpretation. Where formulation details were unavailable in the included studies, contraceptive use should be regarded as a potential source of residual endocrine heterogeneity rather than as an adequately controlled hormonal variable.

Hormonal status is a key physiological variable but has often been overlooked. Sex hormones (oestrogen, progesterone, testosterone) modulate cortical excitability and plasticity, so they likely influence tDCS effects. Very few studies have profiled hormones: one well-controlled study drew blood each session and explicitly confirmed women’s estradiol levels in presumed “low” vs. “high” phases. Others simply tested women in a fixed cycle window (e.g., follicular phase) to reduce variance. Most mixed-sex trials, however, did not monitor cycle phase or contraceptive use, effectively averaging across hormonally heterogeneous women. This could obscure or confound sex differences (e.g., a null finding may hide opposite effects in follicular vs. luteal phases). Similarly, no tDCS study to date has examined transgender or nonbinary individuals, or considered gender identity and hormone therapy, which limits generalisability beyond cisgender samples.

Researchers should record and, if possible, control for hormonal status. At minimum, menstrual cycle phase (or menopausal status) and contraceptive/hormone medication use should be logged. Ideally, hormone assays (e.g., estradiol, progesterone) should be taken concurrently with stimulation to verify physiological state. Analyses should test for hormone-by-tDCS interactions, and sample sizes should be powered to detect such effects. In mixed-sex samples, consider analysing women separately by phase or hormone level. Finally, including measures of other sex-related physiology (e.g., skull thickness from CT or bone density) could help disentangle biological sources of variability.

### 5.5. Outcome Measures and Statistical Power

Behavioural and clinical outcomes in tDCS studies are notoriously variable, and sex differences are often small. Many published studies report no significant sex × stimulation interaction, which could reflect low power. For instance, one cognitive-training trial (N = 69) tested sex effects but may have been underpowered to detect modest differences. Indeed, even when a sex difference is observed, it may depend on condition: in one model, F3/SO stimulation yielded no sex gap, whereas CP5–Cz did. Behavioural tasks themselves introduce noise (learning effects, fatigue, motivation). For example, women were faster and more accurate on a motor task at baseline, so differential tDCS gains must be interpreted against these initial disparities. Without adequate sample size or within-subject designs, it is hard to distinguish true sex-modulation from chance or baseline differences.

Future studies should conduct formal power analyses for sex-related hypotheses (e.g., expecting small-to-moderate interactions) and aim for larger N. Within-subject or crossover designs (each subject has its own control) can improve sensitivity to subtle differences. All analyses should include sex × stimulation interaction terms and report effect sizes. Multiple comparisons (e.g., many ROIs or tasks) should be corrected, and null findings for sex effects should be transparently published. Finally, pre-registering protocols and sharing data will allow meta-analytic aggregation, helping to clarify whether observed sex differences replicate across contexts.

### 5.6. Sex vs. Gender Operationalisation and Reporting Standards

A recurring limitation is that many studies operationalise group differences as “sex” (male/female) while implicitly invoking broader “gender” mechanisms (behavioural, psychosocial, experiential) without measuring them directly. This creates interpretive ambiguity: observed group differences in outcomes could arise from (i) biological factors (skull composition, tissue volumes, endocrine state), (ii) gender-linked behavioural factors (task strategy, fatigue tolerance, expectancy), or (iii) their interaction. In addition, several studies interpret group differences as neural “responsiveness,” even though the underlying delivered dose can differ systematically by sex because anatomy and montage jointly shape intracranial fields.

Adopting standardised reporting frameworks would reduce ambiguity: (a) clearly distinguish “sex assigned at birth,” “gender identity,” and “hormonal status,” and report how each was measured; (b) preregister whether the hypothesis concerns biological sex effects on dose (electric field/current density) versus gender-linked differences in behaviour/experience; (c) include minimal demographic/physiological descriptors that are directly relevant to current flow (e.g., head size proxies, skull metrics where feasible).

### 5.7. Peripheral Sensation, Tolerability, and Blinding Integrity as Sex-Linked Confounds

Sex differences in stimulation sensation and blinding can bias behavioural and clinical endpoints via expectancy/placebo/nocebo pathways. This is especially salient at higher intensities: participants’ condition-guess accuracy declines and misclassification patterns change as intensity increases, while sensation severity rises with intensity and shows sex-by-intensity interactions. Separately, modelling work cautions that some montages may produce effects partly through cranial nerve/scalp stimulation rather than consistent cortical dosing, with the additional concern that females may experience proportionally more scalp current/discomfort because less current reaches the brain under comparable settings. Studies testing sex moderation should (a) report full tolerability distributions by sex (not only mean ratings), (b) formally test blinding success by sex and intensity, and (c) consider “active control” conditions that match peripheral sensations more closely. Where feasible, incorporate objective peripheral measures (skin temperature, impedance stability) and model the scalp field distribution alongside brain dosing to separate peripheral vs. cortical contributions.

### 5.8. Dose Individualisation and “Equalising the Brain Dose” Across Sexes

A major limitation of many sex-comparison studies is the assumption that identical device settings (e.g., 2 mA) imply comparable neural dosing. Modelling work shows that this assumption fails. Montage and anatomy can yield large between-person spreads, as well as sex-dependent differences. Emerging evidence suggests that individualised-dosing approaches can improve behavioural outcomes and reduce variability relative to fixed-dose stimulation. These approaches may also reveal more nuanced sex interactions in response patterns. Future trials should directly compare fixed-dose designs versus dose-targeted designs, in which current is adjusted to achieve a matched cortical E-field/current density at the target (using individualised head models). Such designs would allow a clearer test of whether apparent sex differences reflect true neural sensitivity or simply dose differences that masquerade as sex effects.

### 5.9. Temporal Dynamics and Mechanism: Sex Differences May Be More About Time-Course than Peak Effects

Several studies suggest that sex differences in tDCS effects may be expressed in their temporal evolution rather than in simple post-minus-pre endpoints. For example, in MEP paradigms, cathodal inhibition during stimulation and its after-effects can be stronger and longer-lasting in women. In contrast, anodal effects may look broadly similar at matched time points. Another report emphasises prolonged inhibitory after-effects in women following cathodal stimulation, indicating divergent time-course profiles across sexes. When studies rely on a single post-stimulation time point, these dynamics may be missed, leading to misclassification of sex effects as absent or inconsistent. Future studies should incorporate dense sampling with multiple post-stimulation measurements. They should also model sex differences in response trajectories using growth-curve or mixed-effects approaches rather than relying on single endpoints. Mechanistically, combining time-course assays (MEPs/EEG) with behavioural tasks may clarify whether sex differences in physiology predict the timing and persistence of behavioural changes.

### 5.10. Clinical Translation Limits: Nonrandomised Treatment Allocation and Baseline Imbalances

Clinical datasets provide valuable scale, but causal inference is limited when treatment assignment is nonrandomised. In a large tinnitus cohort, treatment groups were clinic-selected (profile/preferences), raising selection-bias concerns even if sex was not used in assignment. Sex distributions were uneven across therapies (e.g., HD-tDCS group heavily male), and baseline symptom severity often differed by sex. Although mixed models can partially adjust for confounders, residual confounding may inflate apparent sex-by-treatment differences.

For clinical translation, future studies should prioritise randomised or stratified-randomised designs that balance sex and key baseline prognostic factors. Where observational designs are unavoidable, future studies should adopt stronger causal inference methods, including propensity weighting and doubly robust estimation. Studies should also explicitly test whether sex moderation persists after covariate balancing.

### 5.11. Task-Specificity and the Risk of Overgeneralising “Sex Differences in tDCS”

Across studies, sex moderation is often task- and polarity-specific rather than general. For example, sex differences in training gains may emerge under anodal stimulation during training but not under cathodal or sham conditions, and may not persist at later follow-up. Similarly, physiological sex effects may be more pronounced for inhibitory (cathodal) plasticity than for anodal facilitation. Treating “sex effects” as universal across outcomes risks generating contradictory conclusions across studies and laboratories. Instead, sex moderation should be interpreted as conditional on multiple factors, including polarity, montage, cortical target, outcome domain, and timing of measurement. Future research should predefine a limited set of primary endpoints and use hierarchical modelling approaches to reduce false positives while still capturing cross-domain patterns.

### 5.12. Ageing, Atrophy, and Sex-by-Age Interactions in Delivered Dose

Sex effects on dosing and responsiveness may not be stable across the lifespan. Modelling work indicates sex differences in target current can emerge specifically in older adults, with mediation by atrophy-related metrics (e.g., CSF/GM ratios) and morphology changes. This implies that “sex differences” observed in young samples may not generalise to older or neurodegenerative populations where CSF expansion and brain torque may alter current flow. Future tDCS studies should be powered to test sex × age interactions and should incorporate anatomical mediators (CSF/GM ratio proxies, atrophy indices) into both modelling and statistical analyses. In clinical populations, combine structural imaging with individualised dosing to maintain comparable target stimulation across heterogeneous ageing trajectories.

**Figure 10:**
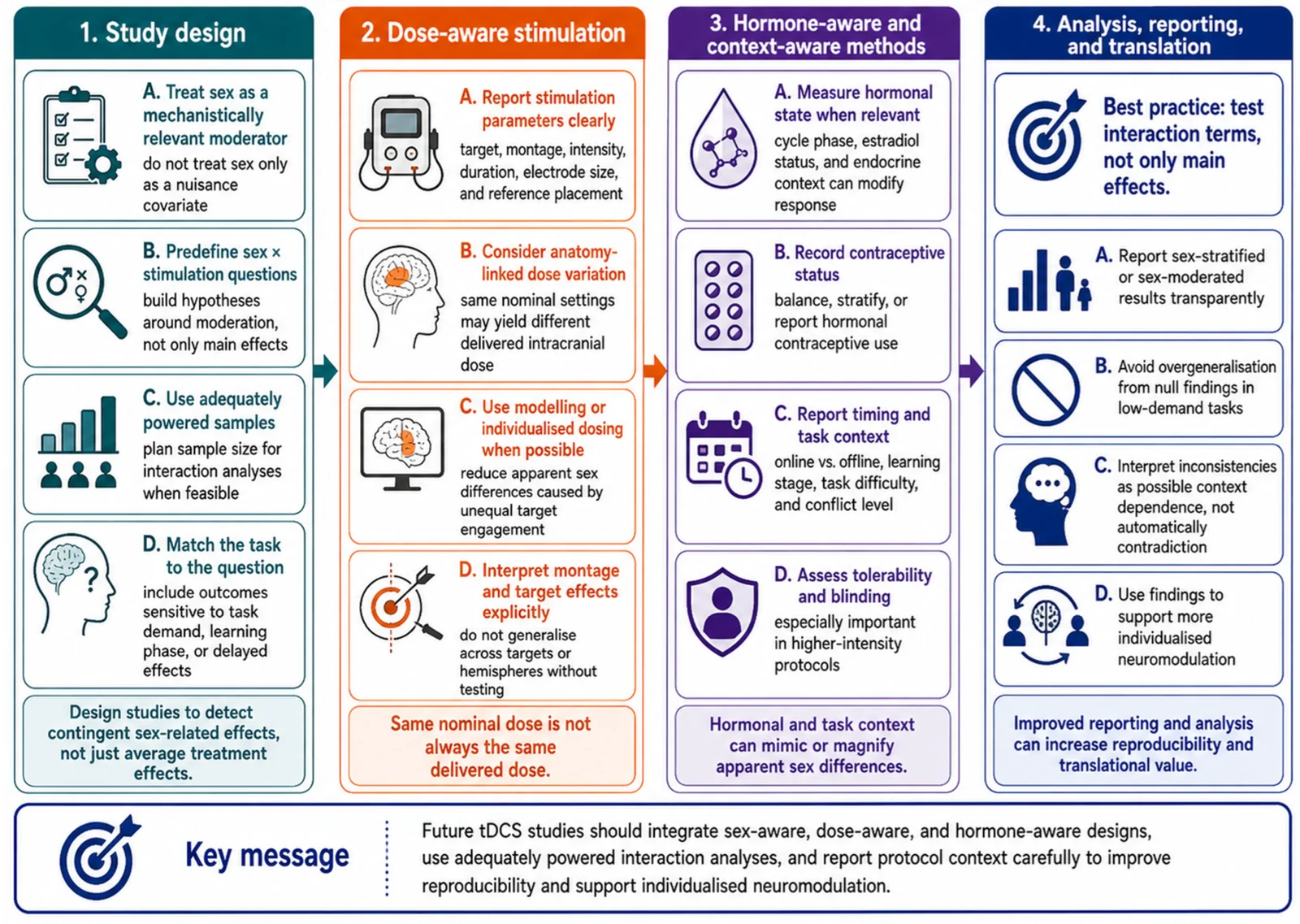
Practical recommendations for future sex-aware tDCS studies. The figure translates the reviewed evidence into methodological recommendations. Future studies should treat sex as a mechanistically relevant moderator, predefine sex × stimulation hypotheses, use adequately powered interaction analyses, report stimulation parameters clearly, consider modelling or individualised dosing when possible, measure or report hormonal and contraceptive status when relevant, and describe task context, timing, tolerability, and blinding procedures transparently. These recommendations aim to improve reproducibility, reduce unexplained heterogeneity, and support more individualised neuromodulation approaches.

The practical implications of the reviewed evidence are summarised in Figure 10, which presents recommendations for future tDCS studies, including sex-aware study design, dose-aware stimulation, hormone- and context-aware methods, and transparent analysis and reporting of sex-related moderation effects.

## 6. Conclusions

Sex-related differences are an important and underrecognised source of variability in tDCS outcomes. Across the studies reviewed, women and men often differed in behavioural, physiological, and clinical responses to stimulation, but these differences were rarely uniform or global. Instead, sex moderation was typically conditional: it depended on montage, target region, stimulation intensity, task demands, timing of outcome measurement, and the specific metric used.

A central conclusion of this review is that “sex effects” in tDCS are best understood as the product of multiple interacting mechanisms rather than a single male–female difference. In some studies, sex likely acts as a proxy for anatomy-related differences in delivered dose (e.g., skull/CSF geometry and current-flow distribution). In others, apparent sex effects are more plausibly linked to hormonal and endocrine state, which can alter cortical excitability and plasticity susceptibility across time. In still others, sex moderation may reflect differences in sensation, blinding, expectancy, or task strategy, especially under higher-intensity or high-demand conditions.

The reviewed evidence also suggests that sex moderation is often most visible in demanding tasks, late learning phases, delayed after-effects, and interaction-based outcomes, rather than in simple baseline performance or global main effects. This helps explain why findings may appear inconsistent across studies and why null average effects can coexist with meaningful subgroup effects.

Taken together, the findings support a shift in how sex is handled in tDCS research. Rather than treating sex as a secondary demographic covariate, future work should treat it as a mechanistically relevant moderator and explicitly model the pathways through which it may influence outcomes. This includes better characterisation of delivered brain dose, improved measurement of hormonal status and cycle phase, careful monitoring of sensation and blinding, and study designs that are powered to test sex-by-stimulation interactions.

In summary, sex does matter for tDCS; however, not in a simple or universal way. The most useful interpretation is not that one sex responds “more” or “better,” but that sex marks a set of biological and contextual factors that can shape whether, when, and how tDCS effects emerge. Recognising and modelling these factors is likely to improve both the reproducibility of tDCS findings and the development of more precise, individualised stimulation protocols.

## Data Availability

No new data were created or analysed in this study. Data sharing is not applicable to this article.
